# Morphological intelligence for dynamic aerial interactions

**DOI:** 10.1126/sciadv.aeg4557

**Published:** 2026-10-23

**Authors:** Yongkang Jiang, Junjin Li, Shuyu Meng, Yanmin Zhou, Bin He, Jamie Paik

**Affiliations:** ^1^ Shanghai Research Institute for Intelligent Autonomous Systems & State Key Laboratory of Autonomous Intelligent Unmanned Systems & College of Electronic and Information Engineering, Tongji University; Shanghai, 201210, China.; ^2^ Guohao School, Tongji University; Shanghai, 201210, China.; ^3^ Reconfigurable Robotics Lab, École Polytechnique Fédérale de Lausanne, Lausanne 1015, Switzerland.

## Abstract

Biological flyers achieve ecological dominance not through flight alone, but by seamlessly integrating aerial locomotion with forceful physical interactions, including snatching prey, perching on irregular surfaces, and operating across diverse media. Translating this agility into aerial robotics demands reconciling a fundamental conflict: impulsive, high-force actions destabilize the flight platform. Here, we present a bio-inspired unmanned aerial vehicle (UAV) equipped with a mechanically intelligent impulsive limb that resolves this tradeoff through physical decoupling. An asymmetric bistable impulse module delivers a 2.3-fold extension within 70 ms, while a soft-linkage architecture reduces the transmission of impulsive interaction forces to the airframe. For the tested seven-segment configuration, intrinsic geometric tolerance corresponds to a passive vertical accommodation range of approximately 70% of the fully deployed arm length, reducing the need for precise altitude regulation within the tested range. We demonstrate the system’s multimodal capacity through dynamic in-flight snatching, energy-efficient perching, high-impact solid fracture, and cross-medium capture in viscous fluids, establishing a new paradigm for UAVs executing physically demanding tasks in complex environments.

## INTRODUCTION

From precise predation to high-speed perching, birds have mastered the art of dynamic aerial interaction by leveraging a specialized morphological architecture. This biological capability relies on morphological buffering: their limbs function as high-speed extendable buffers, allowing them to instantaneously bridge the distance to a target while their wings remain farther from the contact region. However, replicating this combination of impulsive actuation and compliant buffering in aerial robotics remains a critical challenge (*1*–*5*).

Existing aerial manipulation systems typically face a fundamental trade-off between actuation speed and dynamic stability. Rigid manipulators, characterized by high-stiffness kinematic chains, excel in quasi-static precision but lack the capacity to passively dissipate sudden interaction forces (*6*, *7*). During rapid maneuvers, these loads are transmitted directly to the UAV’s center of mass, leading to severe attitude destabilization (*8*–*12*). Furthermore, the limited reach of these systems compels the UAV to operate within close proximity to the target, where possible interference risks damaging the environment or destabilizing the platform (*13*–*16*). Reducing these aerodynamic and mechanical constraints requires a shift from close-range contact to standoff manipulation.

Consequently, extendable soft structures (*17*–*19*), including soft arms and origami-inspired exoskeletons (*20*–*26*), have emerged as promising alternatives. By incorporating structural compliance and foldability, these designs theoretically allow UAVs to maintain a safer standoff distance while buffering impact energy (*27*–*29*). However, current solutions typically introduce a secondary compromise: they are either constrained by limited response speed (*25*, *30*–*34*), or they lack the instantaneous force output required for dynamic interaction (*24*, *29*, *35*–*39*). Although some deployable manipulators achieve rapid actuation, they require separate actuation systems that come with bulky pneumatic pumps or magnetic driving systems (*40*–*45*), precluding seamless onboard integration in untethered aerial robots. A critical gap therefore remains for a manipulation architecture that simultaneously delivers the ballistic actuation required for time-critical tasks and the compliant mechanical buffer essential for flight safety.

To bridge this gap, we report a bio-inspired impulsive limb that realizes physical decoupling by integrating an asymmetric bistable impulse module with a flexure arm. Guided by a theoretical framework governing nonlinear elastic instabilities and kinematic topology, we shaped the system’s strain-energy landscape for rapid energy release while maintaining structural compliance. This architecture mimics the adaptive buffering of avian legs (*21*, *46*), allowing the limb to reduce the transmission of impulsive forces to the flight platform and provide passive vertical accommodation through its intrinsic geometric tolerance. We validate the system’s versatility through four physically and environmentally distinct scenarios, spanning aerial, solid-contact, and liquid environments (Fig. 1). These results demonstrate that morphological intelligence helps resolve the fundamental trade-off between actuation speed and compliance, expanding the operational boundaries of aerial robots. Additional design details, model derivations, parameter definitions, and experimental characterizations are provided in the Supplementary Materials (figs. S1 to S17, tables S1 and S2, and movies S1 to S7).

**Fig. 1.**
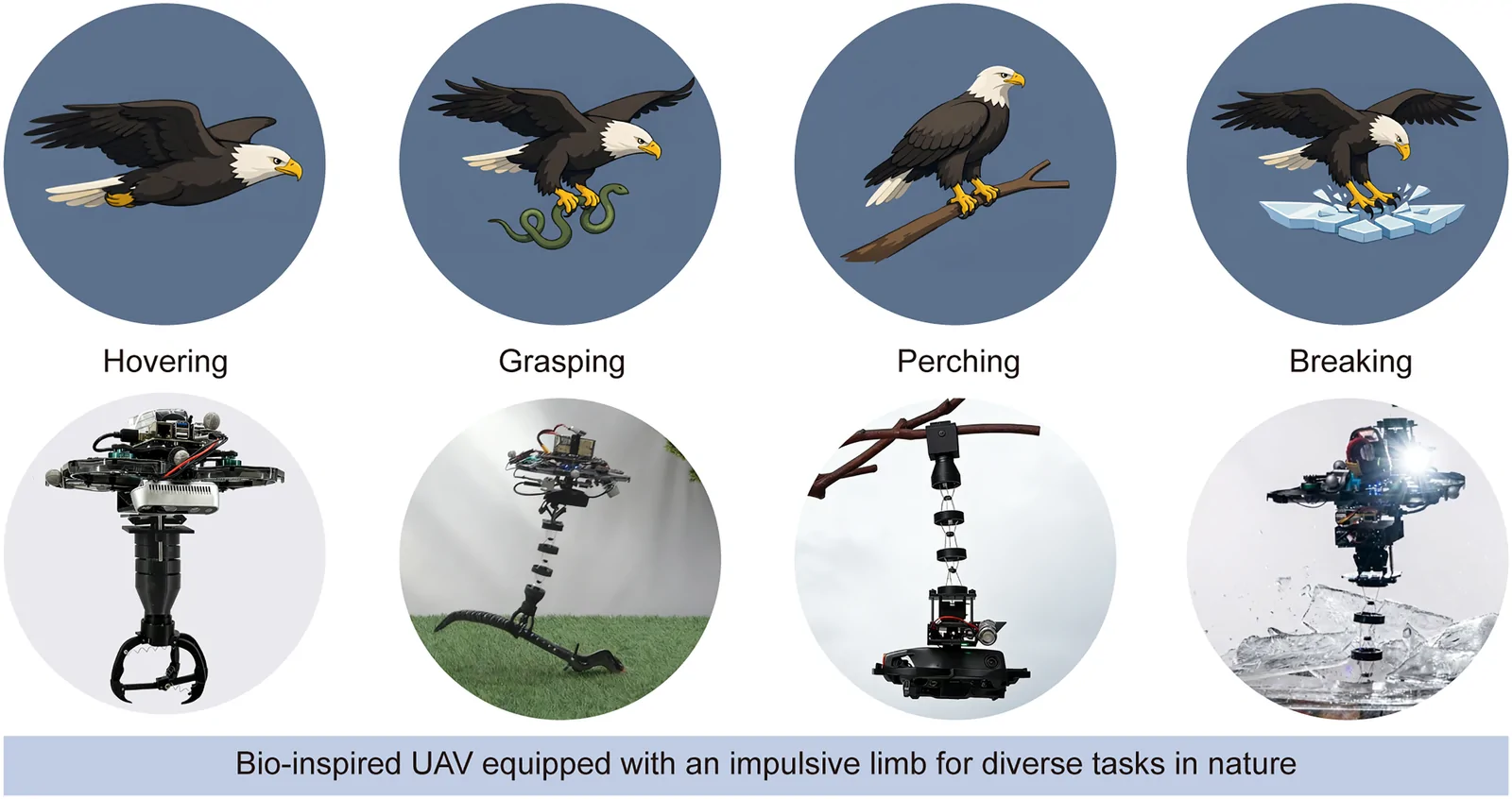
Bio-inspired UAV equipped with an impulsive limb enables morphological buffering for dynamic aerial interactions. Avian predators dynamically alter their limb configurations to execute distinct survival behaviors: maintaining a compact form while hovering, extending talons to grasp moving prey, adjusting grip to perch on uneven branches, and utilizing rapid dashes to break through barriers. The proposed UAV functionally replicates these morphological states. Driven by an asymmetric bistable impulse module, the flexure arm can rapidly deploy and passively adapt its final length upon target contact while simultaneously attenuating impact transients. This morphological buffering enables the UAV to transition from a stable hover to high-impact physical interactions, utilizing passive compliance to reduce the transmission of interaction transients to the airframe.

## RESULTS

### Bio-inspired physical decoupling and impulsive limb architecture

Raptors rapidly extend their legs toward the contact region while keeping their wings and body farther from it. This morphology combines spatial standoff with compliant load attenuation. The distance between the aerodynamic surfaces and the contact region provides the standoff, while limb compliance attenuates contact loads before they are transmitted to the body (*47*). Translating these two functions into aerial robotics requires a system that can rapidly bridge the spatial gap while reducing the interaction load transmitted to the flight platform.

To realize this, we developed an impulsive manipulation system comprising an asymmetric bistable impulse module and a serially connected flexure arm (Fig. 2A). The impulse module stores elastic energy and releases it upon triggering to drive arm deployment. The flexure arm provides an extensible, compliant connection to the end effector and consists of bistable units connected in series through rigid holders. Its deployed reach can be configured by selecting the number of units. The rapid deployment of the arm bridges the gap to the target while allowing the rotor plane to remain farther from the interaction region. After contact, deformation of the flexure arm attenuates the interaction load transmitted to the airframe.

**Fig. 2.**
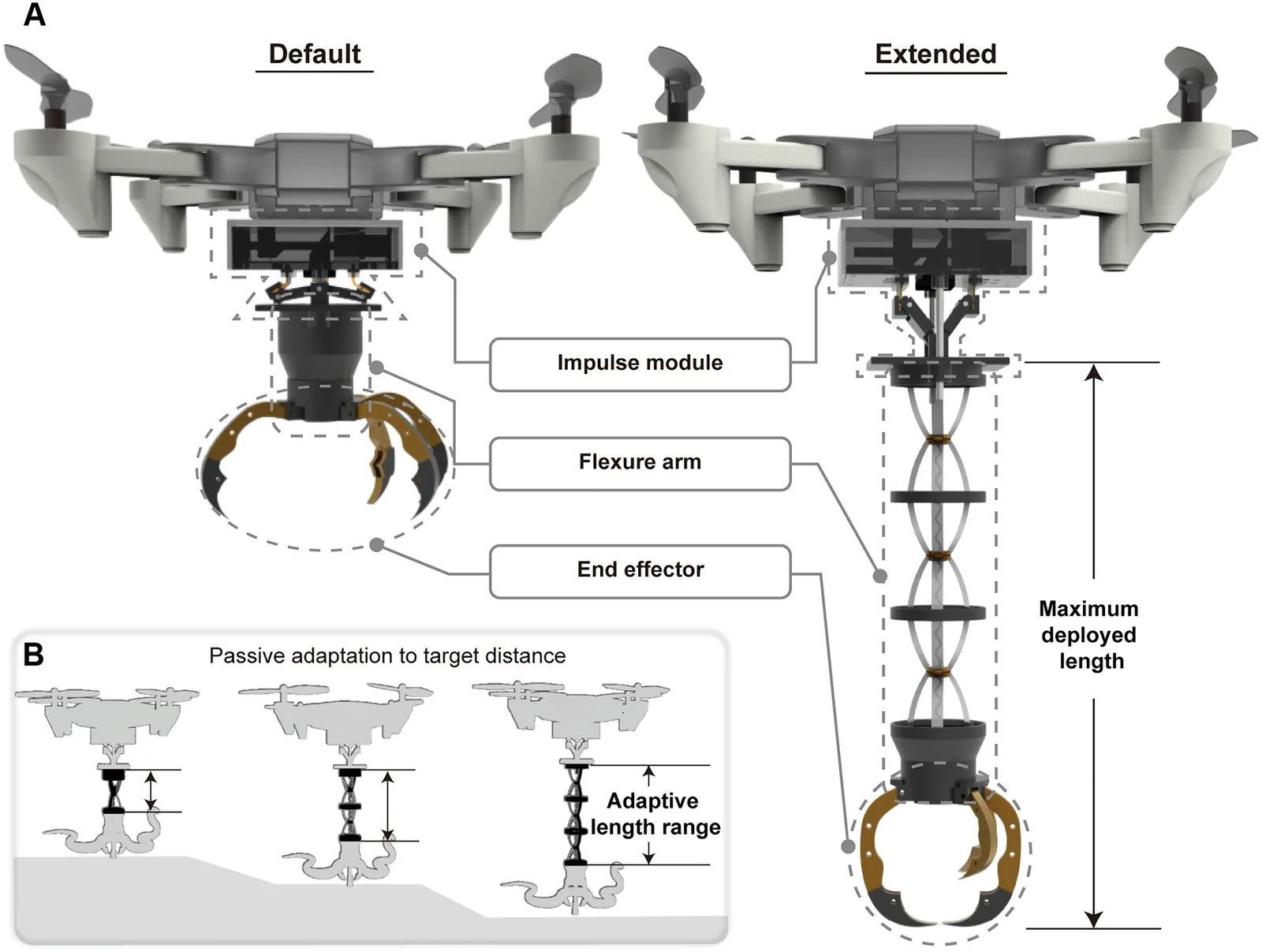
Adaptive morphology of the impulsive limb provides passive height accommodation across variable standoff distances. (**A**) Structural transition of the limb from a compact metastable state to a deployed configuration. Driven by an asymmetric bistable impulse module, the system ballistically projects the end effector for target interaction, mimicking the rapid hindlimb extension of raptors. (**B**) Morphological buffering across different vertical gaps. Triggered by the impulse module, the flexure arm passively adapts its deployed length upon target contact to bridge varying vertical gaps. This mechanism provides a compliant mechanical buffer between the flight platform and the interaction zone while allowing passive height accommodation within the arm’s geometric tolerance.

The flexure arm functions as a mechanical shock filter by deforming during contact and reducing the transient load transmitted to the UAV body. Here, mechanical shock filtering refers to passive attenuation of contact-induced loads and the resulting airframe motion over time. Composed of rigid sheet holders interconnected by flexible bistable units, the arm inherently possesses geometric tolerance. This topology allows the limb to passively adapt its deployed length upon contact and attenuate the interaction transient transmitted to the UAV body. This passive adaptation allows the arm to accommodate different vertical gaps within its measured deployment range without active arm-length regulation (Fig. 2B). The end effector likewise uses target contact to trigger the spring-loaded claws, allowing them to close without a separate gripper command (fig. S11).

To benchmark this performance, we compared the system with existing extensible arms (fig. S1). The comparison shows that the impulse-driven arm exhibits a higher extension rate than the systems included in this comparison. Together with structural compliance, this combination supports the aerial interaction tasks, ranging from high-impact barrier breaking and energy-efficient adaptive perching to the dynamic grasping of moving targets, demonstrating a new paradigm of morphological intelligence in aerial physical interaction.

### Mechanics and design of the asymmetric bistable impulse module

To achieve rapid deployment, we use an asymmetric bistable impulse module that stores elastic energy and releases it rapidly upon triggering. The architecture comprises a rigid base frame and flexure beams that function as the primary elastic storage elements (Fig. 3A). Distinct from symmetric designs, the imposed geometric asymmetry is engineered to decouple total energy capacity from the activation threshold. This configuration confines the mechanism in a loaded metastable default state, enabling a low-force activation that rapidly releases the accumulated elastic energy upon command.

**Fig. 3.**
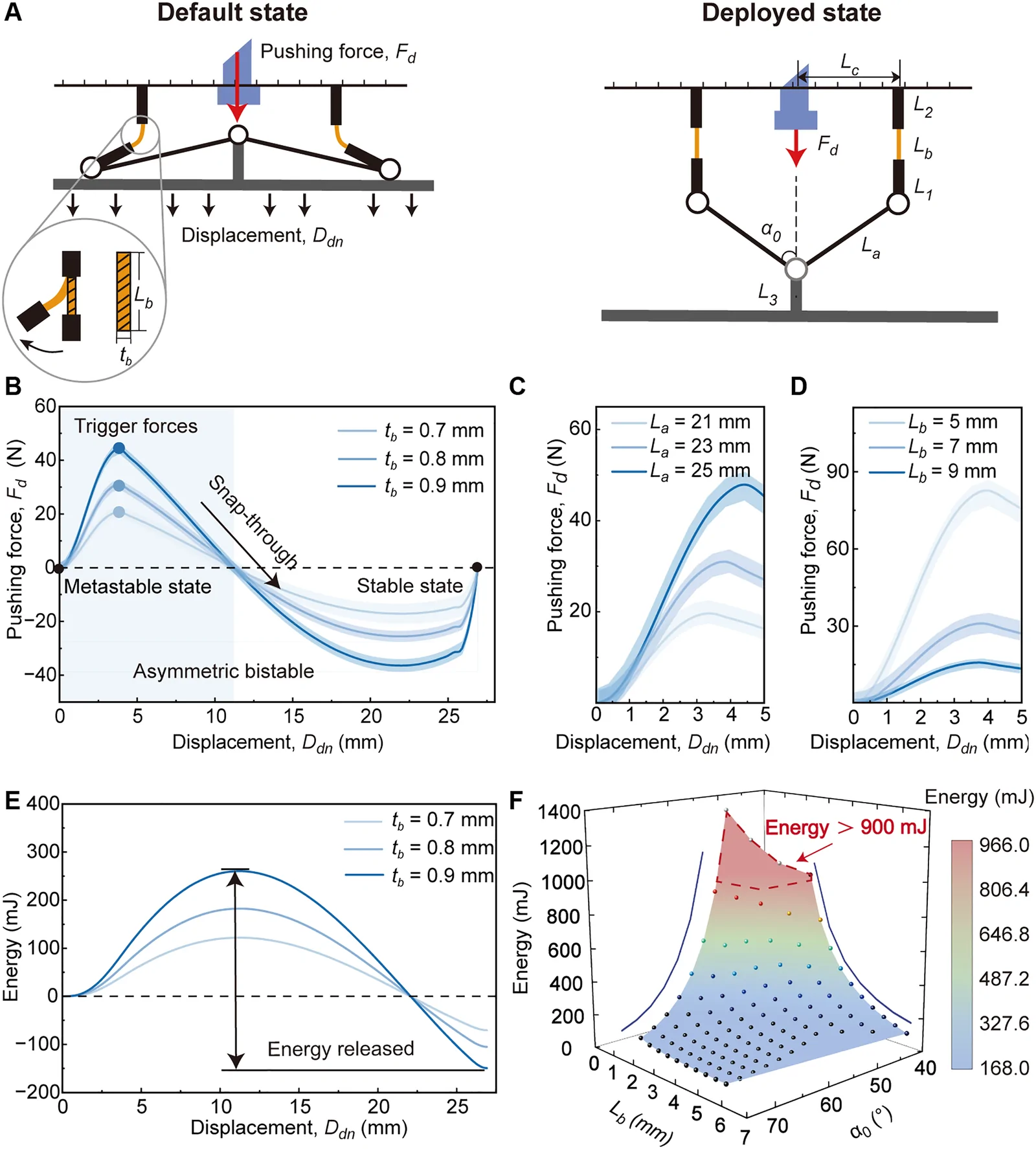
Mechanics and geometric design of the impulse module. (**A**) Modeling of the impulse module in its default (metastable) and deployed (stable) states. A simplified link-spring model defines key geometric parameters for quasi-static analysis: flexure beam length ($L_{b}$), thickness ($t_{b}$), and linkage dimensions. (**B**) Force-displacement profiles characterizing the snap-through instability. Increasing beam thickness amplifies the energy barrier, illustrating the trade-off between energy capacity and the required trigger force. (**C** and **D**) Dependence of the pushing force on link length ($L_{a}$) and flexure beam length ($L_{b}$). (**E**) Strain energy profiles showing that increasing beam thickness enhances energy release during the state transition. (**F**) Energy landscape correlating released energy with beam length and inclination angle ($\alpha_{0}$). [In (B) to (D), solid lines indicate analytical model predictions, and shaded bands represent standard deviation across three independent tests].

The complete operating sequence of the asymmetric bistable impulse module is shown in fig. S10. Starting from stable state 1, the motor drives the horizontal slider toward the critical state. This motion progressively deforms the flexure beams and stores elastic strain energy in the mechanism. Trigger release then initiates snap-through to stable state 2, rapidly releasing the stored energy and producing the impulse required for arm deployment. The motor therefore provides gradual positioning and energy storage, while the rapid deployment is generated by the bistable instability. The resulting impulse drives the serial flexure arm, whose rigid holders transmit kinetic energy between successive bistable units to produce cascading deployment. During reset, the motor-driven cable returns the impulse module to the critical state, followed by reverse snap-through to stable state 1.

Designing this impulse module requires reconciling the trade-off between maximizing energy density and adhering to onboard actuation limits. We established a quasi-static theoretical model (detailed in the Supplementary Materials) to map the mechanical and energetic responses across varying linkage parameters. Within the initial metastable state, the flexure beam (length $L_{b}$, thickness $t_{b}$) undergoes compressive bending, accumulating elastic strain energy. The induced curvature is dictated by structural variables, primarily the link length ($L_{a}$) and the terminal deployment angle ($\alpha_{0}$) (Fig. 3A). The system’s asymmetric bistability is further tuned by adjusting the stopper length ($L_{3}$), which regulates the vertical offset at the plate intersection.

We evaluated the force-displacement and energy profiles during the transition from the metastable to the stable state to map the boundary conditions for energy maximization. Force characterization indicates that increasing the flexure beam thickness $t_{b}$ from 0.7 mm to 0.9 mm elevates the peak trigger force from 20 N to over 45 N, substantially increasing the actuation demand on the motor (Fig. 3B). Sensitivity analysis of the trigger force with respect to $L_{a}$ and $L_{b}$ reveals a strong geometric dependency (Fig. 3, C and D). For instance, decreasing $L_{b}$ to 5 mm increases the peak force to approximately 85 N. Energy profiles in Fig. 3E indicate that increasing the beam thickness from 0.7 to 0.9 mm amplifies the total released energy to approximately 2.5 times. The modeled energy landscape involving $L_{b}$ and $\alpha_{0}$ identified a geometric region in which the modeled energy release exceeded 900 mJ (Fig. 3F). To bypass the onboard actuation limits without compromising energy output, we adjusted the initial position of the vertical slider to preload the impulse module closer to the critical snap-through instability threshold, following the actuator-level geometric preloading strategy (*48*). This geometric offset reduces the required trigger force while retaining the modeled energy release profile.

### Kinematic topology and energy landscape of the flexure arm

To place structural compliance within the load-transmission path, we developed a flexure arm constructed from a serial array of n bistable soft units, where n denotes the total number of flexure-arm segments (Fig. 4A). The deployed limb increases the distance between the rotor plane and the contact point, while the flexure arm places compliance between the end effector and the UAV body. During contact, deformation of the arm attenuates the interaction load transmitted to the airframe.

**Fig. 4.**
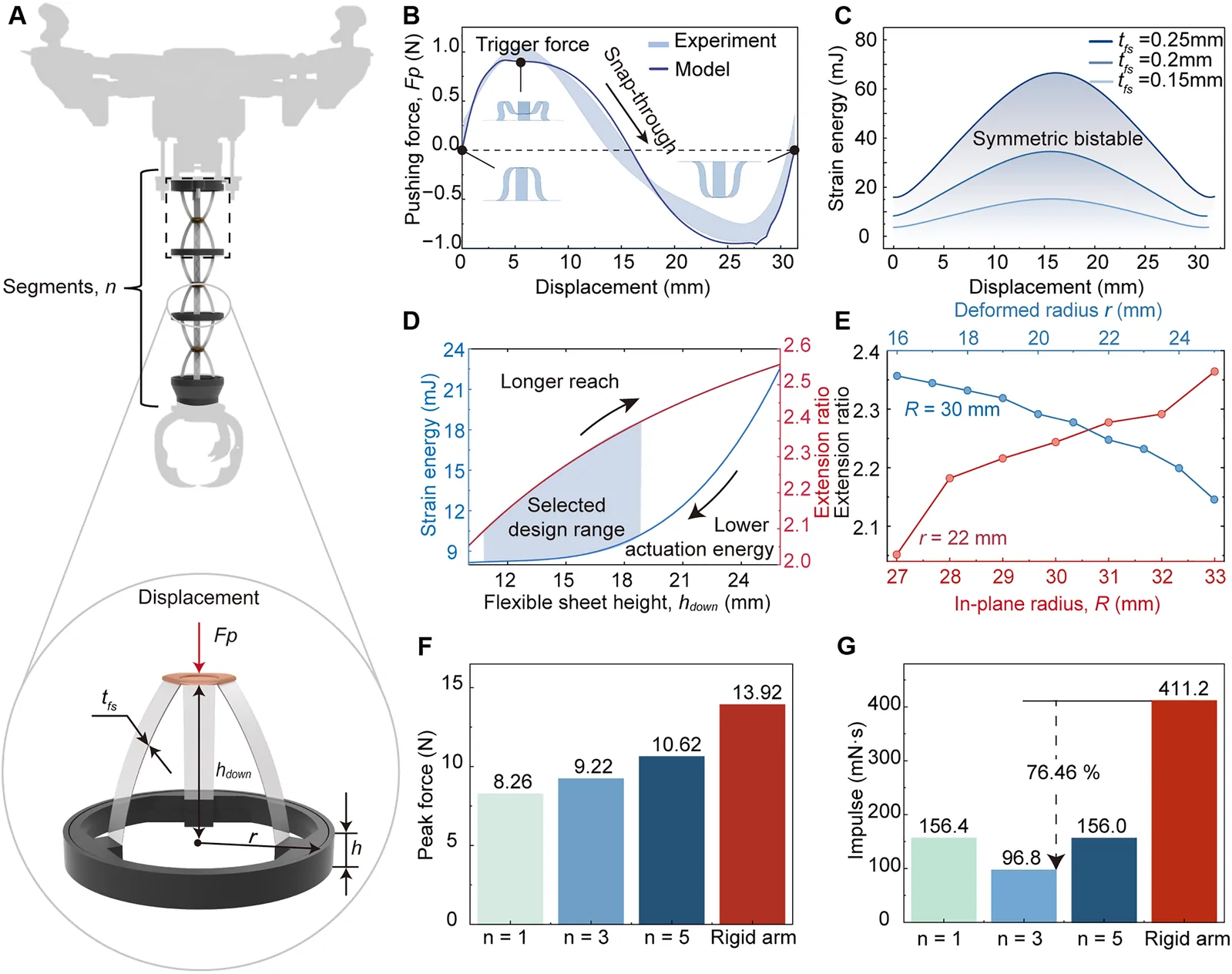
Kinematic topology and energy landscape engineering of the flexure arm. (**A**) Design and geometric parameters of the modular unit. Pre-stressed PET flexible sheets with an in-plane radius R and thickness $t_{\mathrm{fs}}$ are constrained within rigid annular holders with a deformed radius r. $F_{p}$, h, and $h_{\text{down}}$ denote the pushing force, holder height, and lower flexible-sheet height, respectively. (**B**) Force-displacement response characterizing the snap-through instability. The discrete bar-and-hinge model reproduces the overall bistable transition observed in the experimental force-displacement curve. (**C**) Strain energy profiles across varying sheet thicknesses ($t_{\mathrm{fs}}$), defining tunable double-well potentials that govern the trade-off between structural stiffness and deployment resistance. (**D** and **E**) Geometric design. (D) Dependence of the required snap-through strain energy (blue) and extension ratio (red) on the flexible sheet height ($h_{\text{down}}$). The shaded region indicates the selected design range (extension ratio ∈ [2.1, 2.4]), balancing deployment energy and spatial reach. (E) Effects of varying the deformed radius r (fixed *R* = 30 mm, blue) and the in-plane radius R (fixed *r* = 22 mm, red) on the extension capability. (**F**) Peak reaction force transmitted to the UAV body for flexure-arm configurations with n = 1, 3, and 5 and for the rigid arm. (**G**) Contact impulse integrated over the first 100 ms following impulse-module triggering for the same configurations. At n = 3, the flexure arm reduced the impulse over this interval by 76.46% relative to the rigid arm. [In (B), the solid line represents the analytical model, and the shaded band represents standard deviation across three independent tests].

Each structural unit is formed by constraining a laser-cut polyethylene terephthalate (PET) flexible sheet with an in-plane radius R into a rigid annular holder with a deformed radius r, where R>r. For a fixed sheet topology, material, thickness, and clamping condition, the radial mismatch between R and r determines the resulting out-of-plane flexible-sheet height $h_{\text{down}}$. This geometric constraint induces a pre-stressed curvature, creating a double-well potential energy profile that maintains the unit in either a compressed or deployed stable state without continuous power input. The alternating rigid-flexible architecture enables the rigid holders to mediate kinetic energy transfer between structural units to drive deployment. Concurrently, the bistable sheets endow the arm with intrinsic compliance, enabling it to adaptively adjust its length upon contact and function as a mechanical shock filter between the end effector and the UAV body. The arm’s actuation relies on impulsive deployment coupled with thermal retraction: kinetic energy from the impulse module triggers a cascading state transition for extension, while an internal shape memory alloy (SMA) wire contracts upon heating for recovery.

To rationally design this soft-linkage architecture, we mapped its strain energy landscape using a discrete bar-and-hinge model (*49*, *50*) (detailed in the Supplementary Materials). The total energy of the structure is calculated using Eq. (1), where $k_{s}$ and $k_{b}$ represent the stiffness coefficients, $\delta_{i}$ denotes the axial extension of the $i^{\mathrm{th}}$ bar, and $\theta_{j}$ is the rotation angle of the $j^{\mathrm{th}}$ hinge.

$$U_{\text{total}}=U_{\mathrm{bar}}+U_{\text{bending}}=\frac{1}{2}\sum_{i}k_{s}^{i}\delta_{i}^{2}+\frac{1}{2}\sum_{j}k_{b}^{j}\theta_{j}^{2}$$ (1)

Comparison with experiments on the standard unit (R=30 mm,r=22 mm) shows that the model reproduces the overall nonlinear snap-through transition, including a peak pushing force of approximately 0.85 N (Fig. 4B). Our analysis reveals that the energy barrier governing stability is highly sensitive to sheet thickness ($t_{\mathrm{fs}}$). Increasing $t_{\mathrm{fs}}$ from 0.15 mm to 0.25 mm raises the energy barrier from approximately 10 mJ to more than 65 mJ (Fig. 4C), increasing structural stiffness but also substantially raising the actuation energy required for deployment.

A higher extension ratio increases the operational workspace, whereas deployment requires the activation energy to remain within the available actuation range (Fig. 4D). The model shows that increasing the flexible sheet height increases the extension ratio but also raises the required snap-through strain energy. Based on this trade-off, we selected a geometric range corresponding to an extension ratio between 2.1 and 2.4. Ratios below this range restrict spatial reach, whereas larger flexible sheet heights increase the strain energy barrier and friction between adjacent sheets, which limits full deployment. Finally, evaluating radial constraints (Fig. 4E) reveals that the extension ratio scales positively with the in-plane radius R at constant r and negatively with the deformed radius r at constant R, providing geometric design guidance for increasing the arm reach.

The modular arm also provides a configuration-dependent passive vertical accommodation range because target contact can constrain deployment at different lengths without active arm-length regulation. Under fixed-platform conditions, the seven-segment arm provided a measured passive vertical accommodation range ΔH of 277 mm, corresponding to approximately 70% of its fully extended length of 395 mm (fig. S5 and movie S5).

To quantify the contact load transmitted to the UAV body, we measured the reaction force and impulse for flexure-arm configurations with n = 1, 3, and 5 segments, and the rigid arm was tested as the reference (Fig. 4, F and G, fig. S15, and movie S7). The peak reaction forces were 8.26, 9.22, and 10.62 N for the three flexure-arm configurations, compared with 13.92 N for the rigid arm. The corresponding impulses over the first 100 ms following impulse-module triggering were 156.40, 96.80, and 156.00 mN·s for the flexure arm and 411.20 mN·s for the rigid arm. At the matched contact height, the n = 3 flexure arm reduced the peak reaction force by 33.76% and the impulse over this interval by 76.46% relative to the rigid arm.

To further quantify the resulting airframe response during aerial contact, we compared the rigid arm with flexure-arm configurations of $n_{d}$ = 1, 2, and 3 (fig. S16). Here, $n_{d}$ denotes the number of deployed segments during the experiment. The corresponding peak airframe displacements were 61.57, 7.87, 3.47, and 15.66 mm for the rigid arm and the three flexure-arm configurations, respectively. The rigid arm and the $n_{d}$ = 3 flexure arm were tested from the same initial UAV height. Under this matched condition, the flexure arm reduced the peak displacement by 74.57%. Peak velocity and acceleration were also lower for all three tested flexure-arm configurations than for the rigid arm. These results show that the flexure arm attenuates the contact disturbance transmitted to the UAV.

### Dynamic performance and versatile aerial applications

Integrating impulsive actuation with a soft-linkage architecture reconciles the competing demands of reaction speed, operational reach, and dynamic flight stability. We first characterized the complete actuation cycle (Fig. 5 and movie S4). For the configuration tested in Fig. 5, the arm length was measured between the same upper mounting reference and lower end-effector reference. The length increased from 71 mm in the retracted state to 166 mm in the fully deployed state, corresponding to an extension ratio of approximately 2.3. The impulse module drove the arm to a peak deployment velocity of 2.1 m/s, and the arm completed full deployment within 70 ms. After deployment, the SMA-driven thermal recovery mechanism retracted the flexure arm to its compact state within 440 ms. We then examined how the segment number and payload affected these dynamics. Flexure arms containing n=1, 3, and 5 segments completed deployment in approximately 25, 50, and 75 ms, respectively (fig. S13 and movie S6). For payloads of 10, 30, and 50 g, the measured peak deployment velocities were 1.91, 1.89, and 1.58 m/s, while the corresponding peak retraction velocities were 0.98, 0.69, and 0.46 m/s. The corresponding retraction durations were 0.43, 0.45, and 0.62 s (fig. S14). Together, these measurements characterize the deployment and recovery dynamics across the tested arm configurations and payloads.

**Fig. 5.**
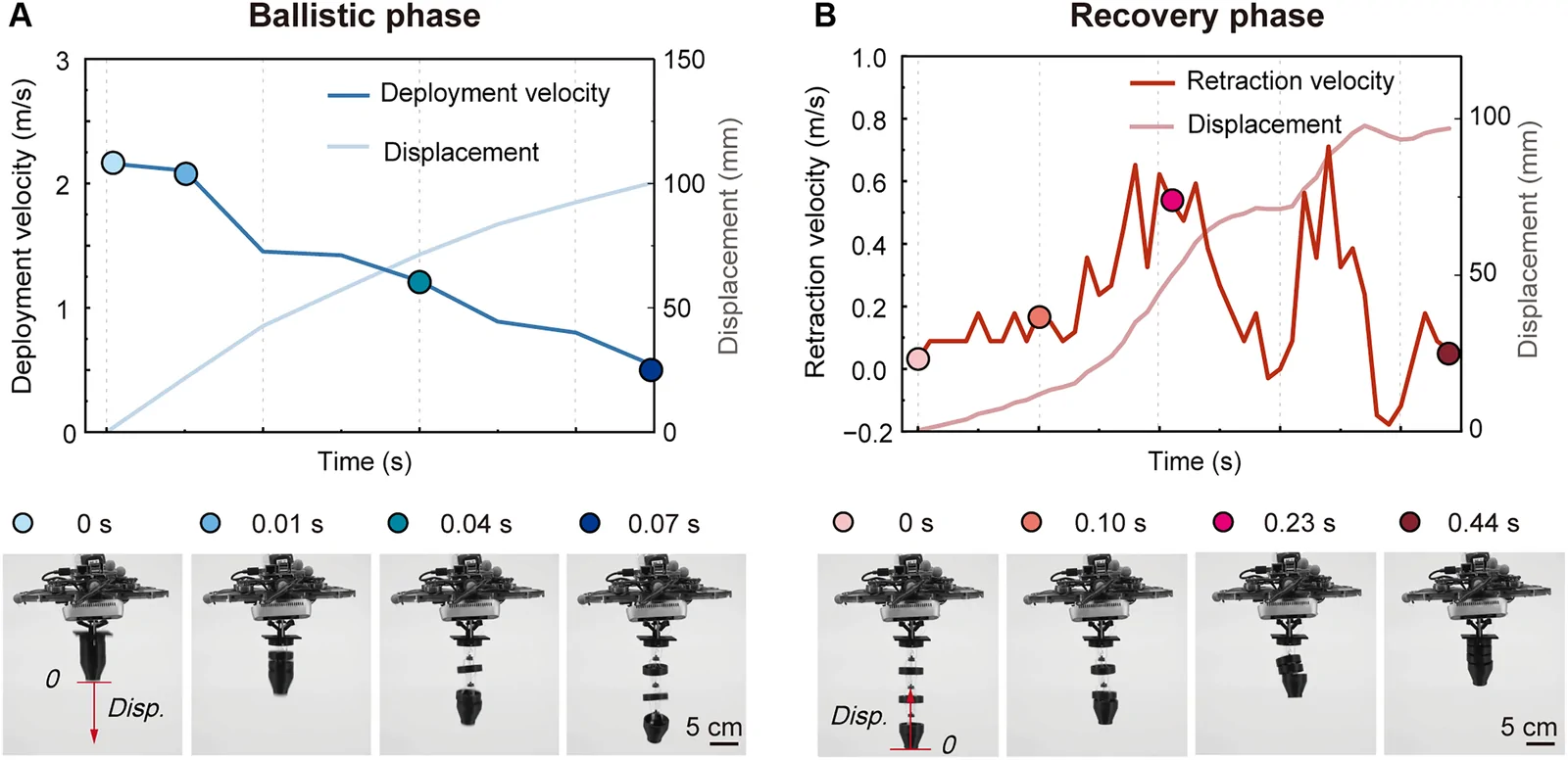
Kinematic characterization of the impulsive actuation cycle. (**A**) Ballistic deployment phase. Triggered by the impulse module, the tested flexure arm reaches full extension in 70 ms with a peak velocity of 2.1 m/s. (**B**) SMA-driven thermal recovery phase. The embedded SMA actuator retracts the flexure arm to its compact state within 440 ms. The reported times characterize the configuration shown here. The effects of segment number and payload are quantified in figs. S13 and S14, respectively.

We next integrated the impulsive limb with target perception and UAV control to evaluate autonomous interception of a moving target, represented here by a snake replica, during flight (Fig. 6B and movie S1). The system combines active UAV positioning and deployment triggering with passive adjustment of the deployed arm length after target contact. Motion capture provided the UAV pose in the world coordinate frame. The target position recovered from the RGB-D measurements was initially expressed in the camera coordinate frame and then transformed into the world coordinate frame using the calibrated camera extrinsics and measured UAV pose. The UAV and target positions were therefore represented in the same world coordinate frame before target prediction and approach control. In Fig. 6A, $p_{U}^{W}$ denotes the UAV position in the world coordinate frame, whereas $p_{T}^{C}$ and $p_{T}^{W}$ denote the target positions in the camera and world coordinate frames, respectively. Detailed position definitions and coordinate transformations are provided in the Supplementary Materials.

**Fig. 6.**
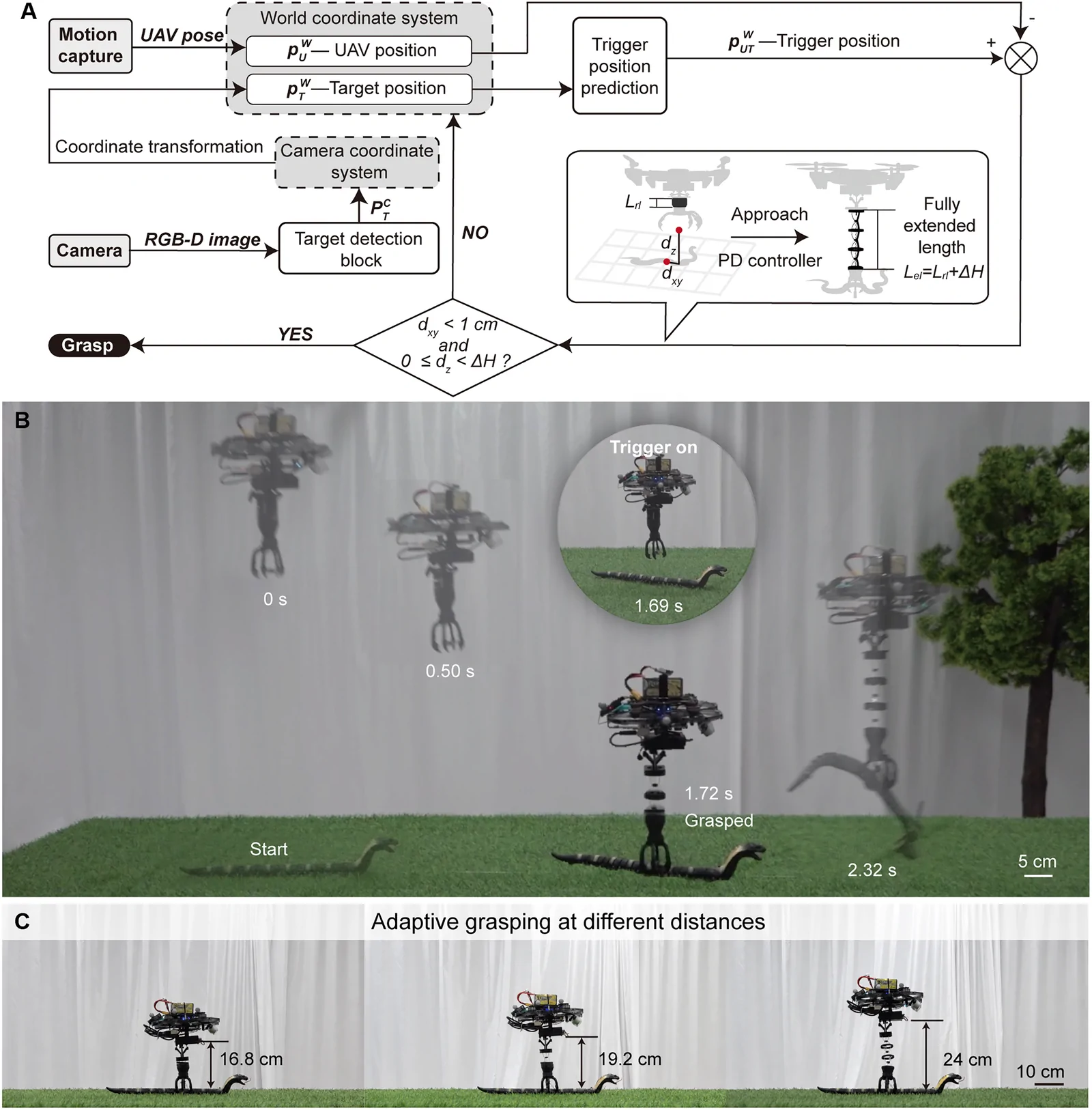
Autonomous target interception with passive arm adaptation. (**A**) Control framework for target detection, position estimation, UAV approach, and deployment triggering. Active control guides the UAV toward the predicted trigger position and activates the impulse module when the horizontal error is below 1 cm and the vertical offset is within the passive tolerance range (ΔH). After triggering, no command regulates the deployed arm length. (**B**) Representative sequence of the UAV autonomously tracking and grasping a moving target. (**C**) Aerial grasping at short, medium, and long distances of 16.8, 19.2, and 24.0 cm between the UAV and target. The same three-segment arm configuration was used in all three experiments. Target contact stopped the arm at different deployed lengths, demonstrating passive adaptation to different target distances.

With the UAV and target positions registered in the same world coordinate frame, a constant-velocity model estimated the target motion from consecutive world-frame target positions and generated the predicted UAV trigger position. During the Approach phase, a three-axis outer-loop PD controller generated the UAV velocity setpoint from the world-frame position error between the predicted trigger position and the measured UAV position. The horizontal components of the velocity setpoint aligned the UAV with the predicted target position, while the vertical component drove the UAV toward the appropriate height above the predicted target. The impulsive limb was activated when the horizontal error $d_{\mathrm{xy}}$ fell below 1 cm and the remaining vertical offset $d_{z}$ satisfied $0\leq d_{z}<\Delta H$. Here, $\Delta H=L_{\mathrm{el}}-L_{\mathrm{rl}}$, where $L_{\mathrm{el}}\;\text{and}\;L_{\mathrm{rl}}$ denote the fully extended and retracted lengths of the flexure arm, respectively. After activation, no command was applied to regulate the deployed arm length. Instead, the arm extended until contact with the target constrained further deployment, allowing its final length to adapt passively to the target height. Using the same three-segment arm configuration, representative trials at vertical separations of 16.8, 19.2, and 24.0 cm showed contact at different deployed lengths (Fig. 6C and fig. S6). Together, the interception and different-distance grasping experiments show that active control positions the UAV and initiates deployment, while target contact passively determines the final deployed arm length within the tested range.

Beyond dynamic interception, the physical decoupling architecture expands the UAV’s operational envelope across three physically demanding, unstructured environments (movie S2). During adaptive perching, an upward-deployed configuration leverages the intrinsic geometric tolerance to passively accommodate variable standoff distances relative to the anchoring branch. The effects of gravity on deployment energy transmission and stability are analyzed in the Supplementary Materials. This mechanism secures the airframe without demanding precise altitude regulation, allowing motor deactivation for persistent monitoring. In high-impact barrier breaching (Fig. 7A), the limb delivers a ballistic impulse to fracture an ice layer. Simultaneously, the flexure arm functions as a mechanical shock filter that attenuates collision transients and reduces the reaction loads transmitted to the airframe. During cross-medium grasping (Fig. 7B), the deployed arm inserts the end effector into the water while the UAV remains above the surface. The increased separation places the rotor plane farther from the water interaction region and supports cross-medium target retrieval. Residual downwash remains visible in movie S2, but the system maintains the standoff distance required to grasp and retrieve the target.

**Fig. 7.**
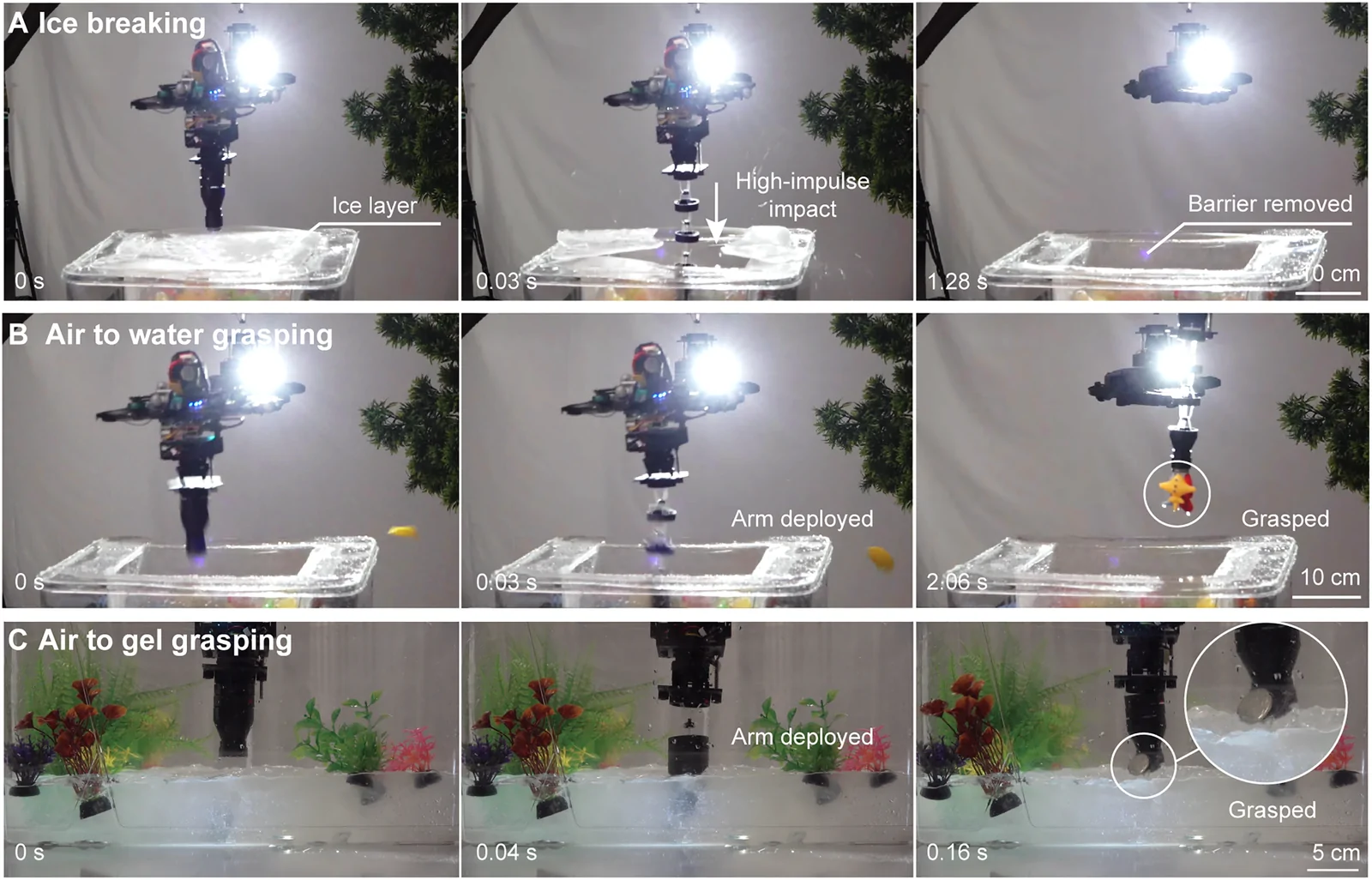
Versatile aerial physical interactions enabled by impulsive actuation and morphological buffering. (**A**) Ice breaking with the impulse module. The instantaneous impact shatters the ice barriers, while the flexure arm functions as a mechanical shock filter that reduces the collision load transmitted to the flight platform. (**B**) Cross-medium retrieval (air-to-water grasping). Leveraging the large deployed extension ratio, the UAV retrieves targets from an increased standoff distance. The increased separation places the UAV farther from the water interaction region while supporting cross-medium retrieval. (**C**) Rapid viscous-medium sampling (air-to-gel grasping). A rapid plunge-retract cycle with a contact time below 0.2 s supports target retrieval from the high-damping medium.

Finally, we challenged the system in viscous media (Fig. 7C and movie S3). The UAV executes a rapid plunge-retract cycle, exploiting the ballistic impulse to penetrate the gelatinous medium and retrieve a target (contact time <0.2 s). The subsequent SMA-driven retraction limits the duration of exposure to fluid drag and surface adhesion, supporting target retrieval from the viscous medium.

## DISCUSSION

The pursuit of dynamic aerial manipulation has long been limited by the fundamental conflict between actuation speed and flight stability. Rigid manipulators afford precision but transmit destabilizing reaction torques directly to the underactuated floating base, whereas soft counterparts offer compliance at the expense of actuation speed. By integrating an asymmetric bistable impulse module with a soft-linkage topology, this design reduces the transmission of interaction transients to the flight platform. This architecture delivers a 2.3-fold extension within 70 milliseconds to rapidly close spatial gaps, while the flexure arm operates as a mechanical shock filter that attenuates impact transients transmitted to the UAV. At the system level, deployment-driven contact couples sequential snap-through, flexure-arm deformation, target contact, and UAV rigid-body motion. The force, impulse, and airframe-motion measurements reported here provide quantitative benchmarks for developing and validating a predictive coupled model across different contact conditions.

Beyond reducing impact transmission, this physical decoupling relaxes the control requirements for dynamic aerial interactions. Conventional high-speed interception often requires accurate altitude regulation near contact. Here, part of the control function is embodied in the flexure arm’s mechanics. For the three-segment configuration used in the aerial flight experiments, the UAV employed a simplified trigger condition based on the arm’s passive vertical tolerance ΔH. After activation, the arm passively adapted its final deployed length without active arm-length regulation.

This paradigm of morphological buffering extends the operational envelope of aerial robots into environments previously precluded by aerodynamic or collision risks. By increasing the distance between the UAV and the interaction region, the system reduces the need for close-proximity operation and provides greater separation from the rotor plane. This capability was validated in energy-efficient perching through passive geometric adaptation, cross-medium retrieval at an increased standoff distance, and high-impact ice breaking. The residual airflow visible in movie S2 indicates that the current standoff distance reduces, rather than completely eliminates, the influence of downwash at the target. The rapid plunge-retract cycle further demonstrates operation in a high-damping medium.

The modular flexure arm architecture allows the working distance to be adjusted by changing the number of segments. Increasing n also changes the deployment distance and duration, required ejection energy, structural mass, and disturbance moment. The required ejection energy is configuration dependent because gravity-assisted cascading and accumulated transmission losses act together. Payload further affects the deployment and recovery dynamics. Because recovery relies on thermally induced SMA contraction, it occurs on a longer time scale than ballistic deployment, and its velocity and duration vary with payload, as quantified in fig. S14. Target contact determines the effective deployment duration by constraining further extension. The post-contact deceleration and transmitted load also depend on the target contact condition. The sheet thickness distribution, holder geometry, and ejection energy should therefore be selected together to maintain reliable deployment and keep the added mass and moment within the payload and stability limits of the UAV. By structurally resolving the trade-off between impulsive force generation and aerodynamic stability, this morphological intelligence shifts the bottleneck of aerial physical interaction from algorithmic control to embodied mechanical design, enabling UAVs to safely execute physically demanding tasks across complex, real-world settings.

## MATERIALS AND METHODS

### Materials and parameters of the prototype

The impulsive limb comprises two primary subsystems: an asymmetric bistable impulse module and a flexure arm. For the impulse module, the rigid frames and sliders were fabricated from polylactic acid (PLA) with a 100% fill density. This maximum density ensures sufficient structural stiffness to withstand the high-impact recoil forces generated during the ballistic snap-through release. The core elastic beams were constructed from polyimide (PI) films, selected for their high fatigue resistance and tensile modulus under repetitive high-strain cycles. The PI beam thickness was constrained to 0.7–0.9 mm to tune the stored strain energy and trigger force of the impulse module.

The flexure arm utilizes a sheet-in-holder topology. The rigid annular holders (PLA) provide kinematic boundary constraints, while the flexible sheets consist of polyethylene terephthalate (PET) films, chosen for their flexibility and low creep characteristics. The fabrication and assembly process of an individual bistable flexure unit is shown in fig. S9. The PET sheet thickness examined in the flexure-unit characterization ranged from 0.15 to 0.25 mm by considering both structural stability and deployment energy. The calculated strain energy increased with sheet thickness and in-plane radius (Fig. 4C and fig. S7), while the configuration-dependent minimum thickness required to resist gravity-induced instability was evaluated in fig. S8A. The configuration-specific sheet dimensions used in the downward- and upward-mounted prototypes are listed in table S1.

### Mechanical characterization and energy profiling

Quasi-static mechanical testing was conducted using a universal materials testing machine (Series 5 F105IM, Mark-10). A motorized linear stage drove the rigid sheet holder to undergo compression and extension at a constant speed of 0.15 mm/s, while recording the reaction force. The strain energy stored in the system was calculated by numerically integrating the force-displacement data. Detailed derivations of the theoretical model are provided in the Supplementary Materials. The force-displacement model was used to calculate the trigger force and strain energy associated with the transition from the compressed state to the deployed state. The predicted response was compared with the experimentally measured force-displacement curve. The model reproduced the overall bistable transition and provided the basis for evaluating the effects of flexible sheet height, sheet thickness, and in-plane radius on the required deployment energy.

Dynamic performance was characterized using a camera operating at 1000 frames per second, recording the complete actuation cycle of the bioinspired impulsive limb (movie S4). Deployment velocity was measured for flexure arms containing n=1, 3, and 5 segments. Deployment and retraction velocities were also measured with payloads of 10, 30, and 50 g. The corresponding retraction durations were determined from the recorded image sequences. Tracking markers were attached to the distal end of the limb, and kinematic data, including position, velocity, and acceleration, were extracted using a motion analysis system (NOKOV).

For the contact comparisons, a rigid reference arm was fabricated by 3D printing with acrylonitrile butadiene styrene (ABS), using the same geometric configuration as the deployed three-segment (*n* = 3) flexure arm. The original impulse module and experimental procedure were retained. During actuation, the output stroke of the impulse module (fig. S10) moved the rigid arm as a fixed-length structure into contact with the target. Reaction loads generated during actuation and target contact were transmitted to the UAV body through the mounting, contributing to the airframe motion recorded in fig. S16.

Reaction loads transmitted to the UAV body were measured using a load cell. The UAV body was connected to the load cell through a height-adjustable platform, while the arm contacted a fixed plane. The flexure arm was tested with n = 1, 3, and 5 segments, and the rigid arm was tested as the reference. The rigid arm and the n=3 flexure arm were tested at the same contact height. The peak reaction force was obtained from each force history, and the contact impulse was calculated as $J=\int_{0}^{t_{\text{imp}}}F_{r}\left(t\right)\mathrm{dt}$, where $F_{r}\left(t\right)$ is the measured reaction force and t=0 corresponds to impulse-module triggering. The integration duration $t_{\text{imp}}=0.1\;s$ was selected because the initial force peaks and rapid oscillations had largely subsided by this time across the tested arm configurations. Airframe motion during aerial contact was recorded for the rigid arm and flexure-arm configurations with $n_{d}$ = 1, 2, and 3. The rigid arm and the $n_{d}$ = 3 flexure arm were tested from the same initial UAV height. The recorded airframe trajectories were used to determine the peak displacement, velocity, and acceleration.

### SMA-driven flexure-arm recovery design

For structural recovery, we integrated an SMA spring with a 1 mm wire diameter and a 4 mm coil diameter. To prevent thermal damage during actuation, the flexure arm connectors feature a hollow-core design. This allows the SMA spring to be routed internally, directly anchoring the proximal and distal ends without physically contacting the polymer structure. To limit prolonged thermal exposure, the system applies a 7.4 V pulse for 0.5 s, followed by immediate deactivation.

### Validation of geometric tolerance and passive adaptation

To characterize passive height accommodation independently of flight control, we conducted fixed-platform, contact-limited deployment tests using a seven-segment flexure arm (fig. S5 and movie S5). After activation, target contact constrained the arm at different deployed lengths without active arm-length regulation. The measured passive vertical accommodation range was 277 mm, corresponding to approximately 70% of the fully extended arm length of 395 mm. These measurements characterize the seven-segment configuration. The dynamic aerial grasping experiments in Fig. 6C and fig. S6 were conducted separately using a three-segment arm.

### Aerial platform integration and system architecture

Aerial experiments were conducted using a custom-designed quadrotor platform powered by brushless motors (2004 PLUS, LANNRC), selected for their low center of gravity and dynamic balance. The impulsive limb was mounted below the fuselage, with its center of mass positioned near the geometric center of the UAV to limit deployment-induced disturbance torque. The integrated UAV and impulsive limb had a measured mass of 0.83 kg, including 144.57 g for the complete impulsive limb assembly. The estimated mass of the UAV body and onboard components was therefore approximately 685 g. A single assembled flexure unit weighed 2.04 g. During the aerial experiments, the system grasped and transported a maximum payload of 450 g. The measured masses of the aerial platform and limb components are summarized in fig. S17.

The avionics architecture centers on a high-performance onboard computer (Orange Pi 5 PRO), which executes visual algorithms and control strategies. Environmental perception relies on a ventral depth camera (RealSense D435i, Intel) for real-time target tracking, supplemented by reflective markers attached to the airframe for pose regulation via a NOKOV motion capture system. A lightweight YOLOv5n model ran on the onboard computer and returned the target bounding box. Reflective markers and a motion capture system provided the UAV pose.

The YOLOv5n detector returned a bounding box in the color image. The center of this bounding box was paired with the aligned depth measurement and back-projected using the camera intrinsics to obtain the target position $p_{T}^{C}$ in the camera coordinate frame. The fixed camera-to-body extrinsic transformation and the measured UAV pose then mapped this position into the world-frame target position $p_{T}^{W}$. Consequently, the UAV position $p_{U}^{W}$ and registered target position $p_{T}^{W}$ were expressed in the same world coordinate frame before entering the prediction and approach-control modules. The target velocity was estimated from consecutive world-frame target positions, and a constant-velocity model predicted the target position over the prediction horizon $T_{p}$, which was set to the fixed mechanism deployment time. The predicted horizontal coordinates and vertical separation $L_{\mathrm{rl}}$ (the fully retracted length of the flexure arm) then defined the UAV trigger position $p_{\mathrm{UT}}^{W}$ to avoid collisions. Once the horizontal alignment error $d_{\mathrm{xy}}$ fell below 1 cm and the residual vertical offset satisfied $0\leq d_{z}<\mathrm{\Delta H}$, the onboard computer sent a grasp command to the microcontroller. The microcontroller triggered the bistable mechanism and initiated rapid limb deployment. No command was applied to regulate the deployed arm length after triggering. Instead, contact with the target constrained further extension, allowing the final arm length to adapt passively to the target height. Detailed position definitions, coordinate transformations, prediction equations, and control laws are provided in the Supplementary Materials.

## Supplementary Materials

### The PDF file includes:

Supplementary Text Figs. S1 to S17 Tables S1 and S2 Legends for movies S1 to S7 Legend for code S1

### Other Supplementary Material for this manuscript includes the following:

Movies S1 to S7

Code S1
