# Supplementary material for: Morphological intelligence for dynamic aerial interactions
Source: Sci Adv. 2026 Oct 23;12(43):eaeg4557. doi: 10.1126/sciadv.aeg4557 (PMC13693677; doi:10.1126/sciadv.aeg4557)
Supplement: Supplementary file 1 [file sciadv.aeg4557_sm.pdf]

Science Advances
AAAS

Supplementary Materials for

Morphological intelligence for dynamic aerial interactions

Yongkang Jiang *et al.*

Corresponding author: Yanmin Zhou, yanmin.zhou@tongji.edu.cn; Bin He, hebin@tongji.edu.cn; Jamie Paik, jamie.paik@epfl.ch

Sci. Adv. **12**, eaeg4557 (2026)
DOI: 10.1126/sciadv.aeg4557

The PDF file includes:

Supplementary Text
Figs. S1 to S17
Tables S1 and S2
Legends for movies S1 to S7
Legend for code S1

Other Supplementary Material for this manuscript includes the following:

Movies S1 to S7
Code S1

Supplementary Text

The geometric and material parameters of the tested flexure-arm configurations are summarized in Table S1, and the symbols used throughout the mechanical models and control framework are defined in Table S2.

Mechanical modeling of the asymmetric bistable impulse module

To analyze the mechanics of the impulse module, we establish a simplified asymmetric model (Fig. S2). The rigid links (length L_1, L_2) clamp the flexible beam (length L_b). The lower rigid link (length L_1) connects to a rigid side plate (length L_a) via a flexible hinge C. The two side plates are coupled at hinge B. Assuming the surrounding elements are strictly rigid, the flexible beam functions as the primary strain energy storage element and remains the focus of this analysis.

At the initial state (Fig. S2, left), Y is the displacement of the end of the flexible sheet along the y-axis, and X is the displacement in the x-axis caused by large deformation. l_0 is the vertical distance between the hinge B and the top plate. We can calculate X using Eq. (S1), and we treat it as a constant here. θ_0 is the initial rotation angle between the lower rigid link and the x-axis at the hinge C. α_1 is the initial angle between the rigid side plate (length L_a) and the x-axis. We approximate the flexible beam's deflected profile using a parabolic function, $x_c = \sqrt{2py_c}$.

$$y_C = \sqrt{L_a^2 - L_3^2} - L_c$$
$$L_b + L_1 = \int_0^{y_c} \sqrt{1 + 2py}dy = \frac{1}{3p}\left[(1 + 2py_c)^{\frac{3}{2}} - 1\right] \qquad (S1)$$
$$l_0 = L_2 + x_C - L_3 = L_2 + \sqrt{2py_c} - L_3$$

The geometry satisfies the following:

$$L_c + L_1 sin\theta_0 + y_0 = \sqrt{L_b^2 - L_3^2} = L_a sin\alpha_1 \qquad (S2)$$

We apply a downward force F_d to the bottom corner of the mechanism. Force equilibrium on the side plates yields:

$$F_d = 2F_b cos\alpha \qquad (S3)$$

Here, F_b is the force on the side plate at the opposite hinge, and α is the angle between the side plate and the center line. Applying the principle of equivalent forces to the free end of the beam (Fig. S2 inset) yields the following loads:

$$F_x = F_b cos\alpha$$
$$F_y = F_b sin\alpha \qquad (S4)$$
$$M = F_b L_1 \sin(\theta_b + \alpha)$$

F_x, F_y, and M are the axial force, shear force, and bending moment on the beam at the free end. To maintain computational efficiency, we adopt a linear superposition approach, incorporating shear effects appropriate for short beams. Specifically, the displacement in the x-

axis comes only from F_x and follows a linear relationship, whereas the displacement in the y-axis comes from both F_y and M as the sum of the displacements caused by each one. Similarly, the rotation angle at the free end comes from both F_y and M, representing the sum of the rotations caused by each load. Let X, Y, and θ_b be the total displacement in the x-axis, total displacement in the y-axis, and total rotation angle. We have:

$$\begin{gathered} M = F_b L_1 \sin(\theta_b + \alpha) \\ Y = Y_{F_y} + Y_M + y_0 = \frac{F_y L^3}{3EI} + \frac{ML^2}{2EI} - \frac{F_y L}{GA} + y_0 \\ \theta_b = \theta_{F_y} + \theta_M + \theta_0 = \frac{F_y L^2}{2EI} + \frac{ML}{EI} + \theta_0 \end{gathered} \tag{S5}$$

Based on the geometry, if the tip displacement is D_{dn}, we have:

$$\begin{gathered} L_a sin\alpha = L_c + Y + L_1 \sin\theta_b \\ D_{dn} = (L_a cos\alpha + L_1 cos\theta_b + L_b - X) - (L_b - x_0 + L_1 cos\theta_0 - L_3) \end{gathered} \tag{S6}$$

For a given $\alpha \in (\alpha_1, \pi - \arcsin(\frac{L_c}{L_a}))$, we can solve Equation (S6) and find F_y, F_x, D_{dn}. Finally, we calculate the stored energy by integrating the force:

$$E_{ejection} = 2\int_0^{D_{dn}} F_d d\xi \tag{S7}$$

Here, $E_{ejection}$ is the energy stored in the mechanism when the tip moves to D_{dn}, compared to the initial state. ξ is the displacement coordinate used in the work integration.

<u>Mechanical modeling of the bistable unit in the flexure arm</u>

To accurately characterize the nonlinear mechanical behavior of the arm unit during the bistable snapping process, the bar-and-hinge model is adopted for theoretical modeling. This approach discretizes the continuous PET flexible sheet into a mesh system comprising rigid links and rotational hinges, effectively transforming the continuum mechanics problem of complex surfaces into a structural mechanics problem.

In this study, a crease-free PET sheet is formed into a pre-stressed bistable configuration via boundary displacement loading (inward compression along three edges). Given the absence of explicit creases in this configuration, the system's deformation energy is primarily dominated by the in-plane stretching and out-of-plane bending of the discretized mesh. Consequently, the total elastic potential energy of the unit is expressed as:

$$U_{total} = U_{bar} + U_{bending} = \frac{1}{2}\sum_i k_s^i \delta_i^2 + \frac{1}{2}\sum_j k_b^j \theta_j^2 \tag{S8}$$

Here, U_{bar} represents the strain energy stored in the tension and compression of the bars, corresponding to the in-plane membrane stress; $U_{bending}$ represents the energy stored in the

rotation of virtual hinges, corresponding to the bending stress of the panel. δ_i denotes the axial elongation of the ith bar, and θ_j denotes the variation in the rotation angle of the jth hinge. The stiffness coefficients are defined as:

$$k_s = \frac{EA_T t_{fs}}{L^2}, \qquad k_b = \frac{EL^2 {t_{fs}}^3}{6(A_1 + A_2)} \tag{S9}$$

where E is the elastic modulus of the material, t_{fs} is the sheet thickness, and L is the characteristic length. A_T refers to the area of a single triangular panel in the discretized mesh for calculating the in-plane bar stiffness of the bar and hinge model. A_1, A_2 represent the areas of two adjacent triangular panels on either side of a bending hinge, which are used to derive the bending hinge stiffness (Fig. S3).

Through convergence calculations, the displacement field of each node at a specified loading step is obtained, allowing for the reconstruction of the instantaneous equilibrium configuration of the bistable unit. By substituting the solved geometric parameters into Eq. (S8), the energy evolution curve for the entire deformation process is derived. The force-displacement response curve of the arm unit can then be predicted by differentiating this energy curve with respect to displacement. Using the discrete bar-and-hinge model, we calculated the strain energy of the bistable flexure unit for PET sheet thicknesses from 0.10 to 0.25 mm and in-plane radii from 27 to 33 mm. The calculated strain energy ranged from 2.41 to 41.92 mJ across the investigated parameter combinations (Fig. S7). The results quantify the increase in strain energy with sheet thickness and in-plane radius and provide the energy basis for selecting the PET sheet geometry used in the prototypes.

<u>Calculation of the extension ratio</u>

To optimize the arm's performance and achieve a high extension ratio, we analyze the geometric parameters governing its macroscopic extensibility. The extension ratio k_e is defined as the ratio of the fully extended length L_{el} to the retracted length L_{rl}:

$$k_e = \frac{L_{el}}{L_{rl}} \tag{S10}$$

Based on the arm structure shown in Fig. S4, we derive the following geometric relationships:

$$\begin{gathered} L_{rl} = nh + h_{end} \\ L_{el} = nh + h_{end} + n\left(h_{up} + h_{down}\right) \\ k_e = \frac{L_{el}}{L_{rl}} = \frac{n\left(h_{up} + h_{down}\right)}{nh + h_{end}} + 1 = \frac{\left(h_{up} + h_{down}\right)}{h + \frac{h_{end}}{n}} + 1 \end{gathered} \tag{S11}$$

Here, h_{down} denotes the resulting out-of-plane height of the assembled flexible sheet. For a fixed sheet topology, material, thickness, and clamping condition, h_{down} is determined by the in-plane radius R and deformed radius r, rather than specified independently.

Consequently, the extension ratio k_e of the arm is governed by the heights of the intermediate sheet holders (h), the end holder (h_{end}), the heights of the upper and lower flexible sheets (h_{up}, h_{down}), and the total number of units (n). In this case, we have $h_{up} \approx h_{down}$ to increase the extension ratio for a given height h_{down}. Additionally, we constrain $h_{end} \geq h_{down}$ so that the end holder can accommodate the flexible arch plate in the default state. The derivation indicates that increasing h_{down} yields a higher extension ratio. However, an excessive arch height elevates the deployment energy threshold and amplifies inter-unit transmission losses, imposing prohibitive actuation requirements on the overall impulse module.

For the arm configuration tested in Fig. 5, the retracted and fully extended lengths were measured between the same upper mounting reference and lower end-effector reference. The measured values were $L_{rl} = 71$ mm and $L_{el} = 166$ mm. The corresponding extension ratio was therefore 2.3.

<u>Calculation of the intrinsic geometric tolerance</u>

To quantify the arm's passive accommodation of target height variations, we define the passive tolerance ratio γ as the fraction of the fully deployed arm length available for passive vertical accommodation. Here, ΔH denotes the additional vertical range accommodated through arm deployment. The passive tolerance ratio is defined as:

$$\Delta H = L_{el} - L_{rl}, \qquad \gamma = \frac{\Delta H}{L_{el}} \tag{S12}$$

For the seven-segment arm characterized under fixed-platform conditions in Fig. S5 and Movie S5, the passive tolerance ratio is:

$$\gamma = \frac{277}{395} \times 100\% \approx 70\% \tag{S13}$$

<u>Target position registration, prediction, and approach control</u>

UAV position in the world coordinate frame

The motion capture system measured the position and attitude of the unmanned aerial vehicle. The flight position estimator expressed these measurements in the world coordinate frame $\{W\}$. The UAV position vector in $\{W\}$ is denoted by p_U^W. The subscripts U and T identify the UAV and target, respectively. Superscripts W, B, and C identify the world, UAV body, and camera coordinate frames. The position vector p_U^W contains the UAV coordinates X_U^W, Y_U^W, and Z_U^W along the three axes of $\{W\}$. The superscript T outside the brackets denotes the vector transpose:

$$p_U^W = [X_U^W, Y_U^W, Z_U^W]^T \tag{S14}$$

Target position in the camera coordinate frame

The ventral RGB-D camera provided a color image, an aligned depth image, and the calibrated intrinsic parameters. For each detected target, the detector returned the bounding-box limits x_{min}, x_{max}, y_{min}, and y_{max} in the color image. The variables u and v denote the horizontal and vertical pixel coordinates of the bounding-box center, respectively:

$$u = \frac{x_{\min} + x_{\max}}{2}, v = \frac{y_{\min} + y_{\max}}{2} \qquad (S15)$$

Let d denote the valid aligned depth at the center pixel. The quantities f_x and f_y are the focal lengths in pixels, whereas c_x and c_y are the principal-point coordinates. Back-projection gives the target position p_T^C in the camera frame $\{C\}$:

$$p_T^C = \left[\frac{(u - c_x)d}{f_x}, \frac{\left(v - c_y\right)d}{f_y}, d\right]^T \qquad (S16)$$

Transformation from camera coordinates to world coordinates

The calibrated camera extrinsics first transformed the target position from $\{C\}$ to $\{B\}$. Here, p_T^B is the target position in the body frame, R_{BC} is the rotation matrix from the camera frame to the body frame, and t_{BC} is the position of the camera origin expressed in the body frame:

$$p_T^B = R_{\mathrm{BC}} p_T^C + t_{\mathrm{BC}} \qquad (S17)$$

The measured UAV pose then transformed p_T^B into the target position p_T^W in the world frame. Here, R_{WB} is the rotation matrix from the body frame to the world frame:

$$p_T^W = p_U^W + R_{\mathrm{WB}} p_T^B \qquad (S18)$$

Substitution of Eq. (S17) into Eq. (S18) gives the complete coordinate transformation:

$$p_T^W = p_U^W + R_{\mathrm{WB}}(R_{\mathrm{BC}} p_T^C + t_{\mathrm{BC}}) \qquad (S19)$$

The UAV and target positions were therefore expressed in the same world coordinate frame for prediction and approach control.

Target prediction and trigger position in the world frame

Let k denote the current sample index, $k-1$ the preceding sample index, and Δt the sampling interval. The target velocity $v_T^{W(k)}$ was estimated from two consecutive target positions in the world frame:

$$v_T^W(k) = \frac{p_T^W(k) - p_T^W(k-1)}{\Delta t} \quad (S20)$$

A constant-velocity model predicted the target position $p_{T,pred}^W$ over the prediction horizon T_p, which was set to the fixed mechanism deployment time:

$$p_{T,\mathrm{pred}}^W = p_T^W + v_T^W T_p \quad (S21)$$

The trigger position p_{UT}^W used the predicted horizontal coordinates and maintained the vertical separation L_{rl} above the predicted target position to avoid collisions. The quantities $X_{T,pred}^W$, $Y_{T,pred}^W$, and $Z_{T,pred}^W$ are the three components of $p_{T,pred}^W$:

$$p_{\mathrm{UT}}^W = \left[X_{T,\mathrm{pred}}^W, Y_{T,\mathrm{pred}}^W, Z_{T,\mathrm{pred}}^W + L_{rl}\right]^T \quad (S22)$$

UAV approach control and grasp triggering

During the approach, at sampling instant k, the world frame position error $e_p^W(k)$ was defined as the difference between the trigger position $p_{\mathrm{UT}}^W(k)$ and the measured UAV position $p_U^W(k)$:

$$e_p^W(k) = p_{\mathrm{UT}}^W(k) - p_U^W(k) \quad (S23)$$

The approach controller applied proportional and derivative feedback independently along the three axes of the world frame:

$$\begin{bmatrix} v_{\mathrm{cmd},x}^W(k) \\ v_{\mathrm{cmd},y}^W(k) \\ v_{\mathrm{cmd},z}^W(k) \end{bmatrix} = \begin{bmatrix} k_{p,x} & 0 & 0 \\ 0 & k_{p,y} & 0 \\ 0 & 0 & k_{p,z} \end{bmatrix} \begin{bmatrix} e_x^W(k) \\ e_y^W(k) \\ e_z^W(k) \end{bmatrix} + \begin{bmatrix} k_{d,x} & 0 & 0 \\ 0 & k_{d,y} & 0 \\ 0 & 0 & k_{d,z} \end{bmatrix} \left(\begin{bmatrix} e_x^W(k) \\ e_y^W(k) \\ e_z^W(k) \end{bmatrix} - \begin{bmatrix} e_x^W(k-1) \\ e_y^W(k-1) \\ e_z^W(k-1) \end{bmatrix} \right) \quad (S24)$$

Here, $k_{p,i}$ and $k_{d,i}$, $i \in \{x, y, z\}$, are the proportional and derivative gains along each axis, respectively. The sampling interval is incorporated into the derivative gains. The resulting velocity command was sent to the flight controller as a velocity setpoint.

The horizontal alignment error d_{xy} was defined as the Euclidean norm of the x and y components of the position difference between p_{UT}^W and p_U^W. The subscript xy selects the horizontal components, and the subscript 2 denotes the Euclidean norm:

$$d_{\mathrm{xy}} = \left\| (p_{\mathrm{UT}}^W - p_U^W)_{\mathrm{xy}} \right\|_2 \quad (S25)$$

The residual vertical offset d_z was calculated from the UAV height Z_U^W, the predicted target height $Z_{T,\text{pred}}^W$, and the vertical separation L_{rl} to avoid collisions:

$$d_z = \left(Z_U^W - Z_{T,\text{pred}}^W\right) - L_{rl} \tag{S26}$$

The grasp command was issued when:

$$d_{\text{xy}} < 1\text{cm} \quad \text{and} \quad 0 \leq d_z < \Delta H \tag{S27}$$

If either condition was not satisfied, target estimation, prediction, and approach control continued.

<u>Deployment energy transmission and orientation-dependent stability</u>

Deployment orientation changes both inter-unit energy transfer and the stability of the deployed arm. During downward deployment, descending holders release gravitational potential energy and assist the cascading transition. During upward deployment, gravity reduces the energy transmitted to subsequent units and may induce reverse snap-through after deployment. We therefore consider the energy-transfer condition and the post-deployment stability criterion within the same framework.

Using the proximal-to-distal indexing shown in Fig. S4, let i denote the position of the deployed bistable sheet for both the downward- and upward-mounted configurations. The mechanical energy delivered to the subsequent unit at position $(i + 1)$ is

$$E_{i+1} = \begin{cases} \eta\left(E_{left,i} + \Delta E_i + W_{g,i}\right), & for\ downward\ deployment \\ \eta\left(E_{left,i} + \Delta E_i - W_{g,i}\right), & for\ upward\ deployment \end{cases} \tag{S28}$$

Here, η is the inter-unit energy transmission efficiency and satisfies $0 < \eta \leq 1$. The term $W_{g,i} > 0$ denotes the magnitude of the gravitational work associated with holder motion during the transition of unit i. Gravity contributes positively during downward deployment and negatively during upward deployment. $E_{left,i}$ is the residual mechanical energy after unit i completes snap-through, and ΔE_i is the energy difference between its two stable states. The stable states are treated as symmetric in the present model, giving $\Delta E_i = 0$. Cascading deployment continues only when E_{i+1} exceeds the activation-energy barrier of the subsequent unit.

For the upward-mounted arm, each deployed flexible sheet must also support the rigid holders distal to it. Here, we use a conservative approximation in which the holders provide the dominant gravitational load and the self-weight of the PET sheets is neglected. If each holder has mass m and g is the gravitational acceleration, the cumulative load on the sheet at position i is

$$G_i = (n + 1 - i)\, m\, g, \quad i = 1, 2, \ldots, n \tag{S29}$$

Here, n is the total number of flexure-arm units in the analyzed configuration. Under this proximal-to-distal indexing convention, the first flexible sheet is the proximal sheet and supports other holders, giving $G_1 = nmg$. The applied load decreases with i because units farther from the mounting point support progressively fewer distal holders. This force criterion below applies to both deployment orientations but has different physical meanings. For downward mounting, it ensures that the flexure arm remains stable in the initial state before deployment. For upward mounting, it ensures that each flexure unit can overcome the cumulative gravitational load and complete snap-through during deployment. The general condition is therefore

$$F_{i,\max} > G_i = (n+1-i)\ m\ g \tag{S30}$$

For the fixed PET material, the critical force $F_{i,max}$ depends on the sheet thickness t_i, the in-plane radius R_i, the clamped radius r_i, and the boundary conditions imposed by the annular holder. The geometry of each unit must consequently satisfy

$$F_{i,\max}(t_i, R_i, r_i) > (n+1-i)\ m\ g \tag{S31}$$

The same condition can be expressed as the following dimensionless stability margin:

$$\frac{F_{i,\max}}{(n+1-i)\ m\ g} > 1 \tag{S32}$$

Units closer to the mounting point support a larger cumulative weight and therefore require a greater critical reverse snap-through force in the upward-mounted configuration. Increasing sheet thickness raises bending stiffness and improves resistance to gravity-induced reversal. However, it also raises the activation-energy barrier and the impulse energy required for deployment. The design evaluation is therefore divided into two coupled checks. First, orientation-dependent energy transmission is evaluated using Eq. (S28), with the sign of $W_{g,i}$ determined by the deployment direction. Second, for the upward-mounted configuration, the deployed geometry must satisfy Eqs. (S29) to (S32) at every segment position. These conditions together balance cascading transmission, actuation demand, and post-deployment stability.

Configuration-dependent design of the flexure arm

The working distance of the flexure arm can be customized according to task requirements. For applications involving rotor downwash, the required interaction distance depends on the rotor diameter, thrust level, flight condition, surrounding environment, and target sensitivity to airflow. Once this distance has been determined, the number of serially connected flexure segments can be selected to provide the corresponding deployed length. The tested seven-segment configuration

reaches a fully deployed length of 39.5 cm. Adding segments increases the working distance and passive accommodation range, but also increases the structural mass, gravitational load, deployment duration, and disturbance moment. The segment number should therefore remain compatible with the payload, thrust, and stability limits of the UAV.

The holder height and flexible sheet thickness must be selected together with the segment number. The holder height affects the compact length and mass of each unit, while the segment position determines the cumulative mass supported by that unit. The required sheet thickness is therefore determined by the local supported load. As shown in Fig. S8A, units closer to the mounting point generally require greater resistance to gravity-induced instability because they support more distal segments.

The impulse energy must also be adjusted when the segment number changes. During downward deployment, gravitational potential energy released by the descending units assists subsequent deployment, while a longer transmission path increases friction, impact dissipation, structural deformation, and inter-unit energy loss. The trends in Fig. S8B therefore reflect the balance between gravity-assisted cascading and accumulated transmission losses. For the $h = 5$ mm configuration, gravity-assisted cascading reduces the required ejection energy as n increases from 5 to 8. As the segment number increases further, the greater cumulative load raises the minimum PET sheet thickness required for gravitational stability. In the implemented design, the PET sheet thickness changes in discrete increments. The minimum t_{fs} increases from 0.10 to 0.15 mm when n_s increases from 8 to 9 (Fig. S8A). Under the proximal-to-distal position index i, $n_s = n + 1 - i$. Because the snap-through energy barrier increases steeply and nonlinearly with sheet thickness, this thickness step raises the required ejection energy for the $n = 9$ and $n = 10$ configurations. Together with accumulated transmission losses, this effect produces the subsequent increase shown in Fig. S8B.

Modular extension by adding segments should be distinguished from geometric variation of individual flexure units. To examine geometric configurability within a limited dimensional range, we fabricated prototypes with end-effector radii of 0.6r, r, and 1.5r. Their deployed lengths were 8.5, 12.8, and 19 cm, respectively (Fig. S12). These prototypes demonstrate fabrication across the tested range. Changes in unit dimensions require the sheet thickness, material properties, fabrication accuracy, gravitational loading, and deployment energy to be reevaluated for the new configuration.

Dynamic measurements further quantified the effects of segment number and payload. Flexure arms containing $n = 1$, 3, and 5 segments completed deployment in approximately 25, 50, and 75 ms, respectively (Fig. S13 and Movie S6). With payloads of 10, 30, and 50 g, the measured peak deployment velocities were 1.91, 1.89, and 1.58 m/s, while the corresponding peak retraction velocities were 0.98, 0.69, and 0.46 m/s. The corresponding retraction durations were 0.43, 0.45, and 0.62 s (Fig. S14). These results show that both deployment and recovery dynamics depend on the arm configuration and payload.

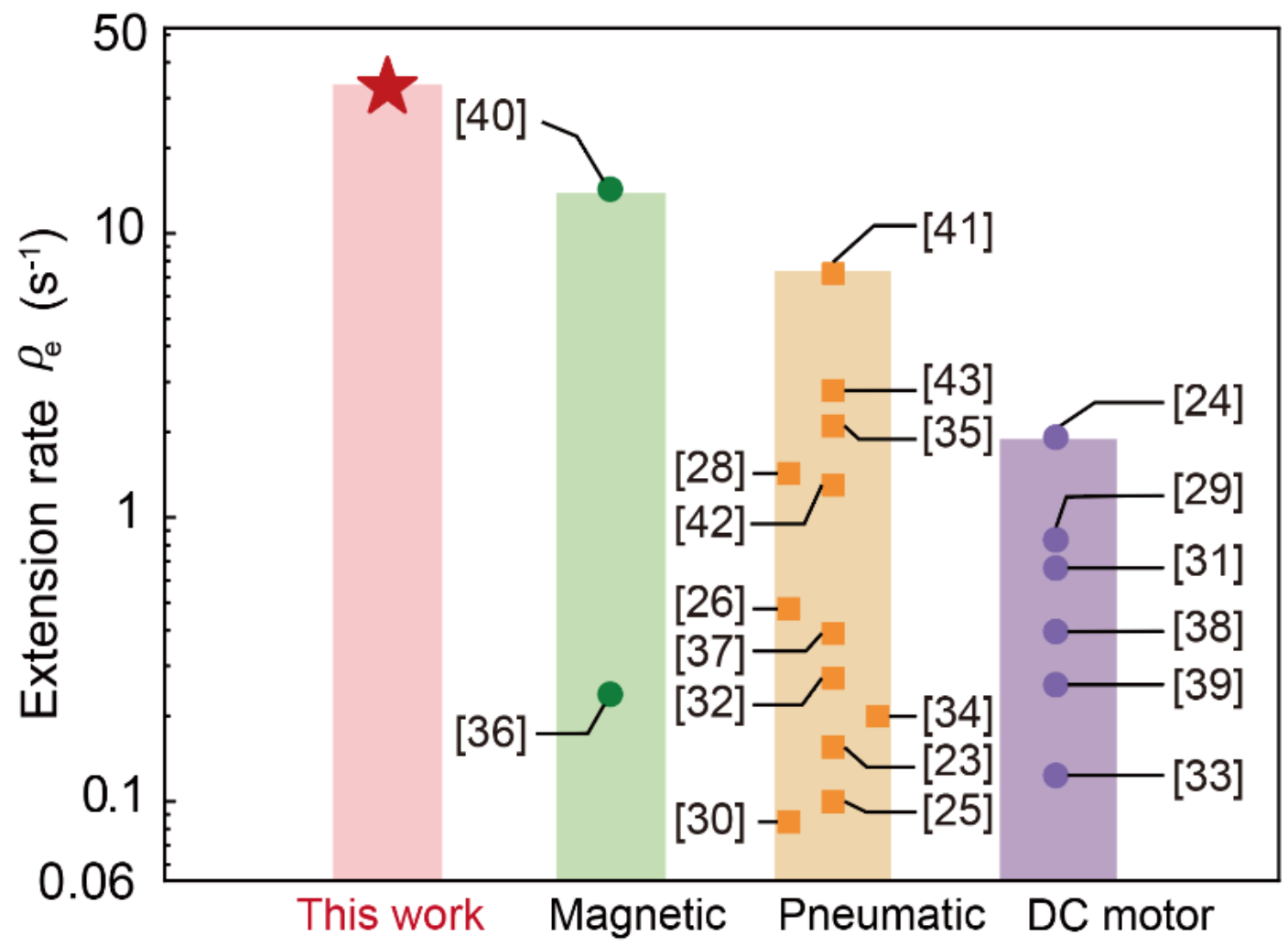

Fig. S1. Deployment performance of extensible arms grouped by actuation method. The vertical axis shows the arm extension rate $\rho_e = k_e/t_d$, where k_e is the ratio of the fully extended length to the retracted length and t_d is the time required to complete deployment. The reported systems are grouped as magnetic, pneumatic, and DC-motor-driven arms, while the proposed flexure arm is shown separately by the red star. Reference numbers are provided next to the corresponding data points. The vertical axis uses a logarithmic scale.

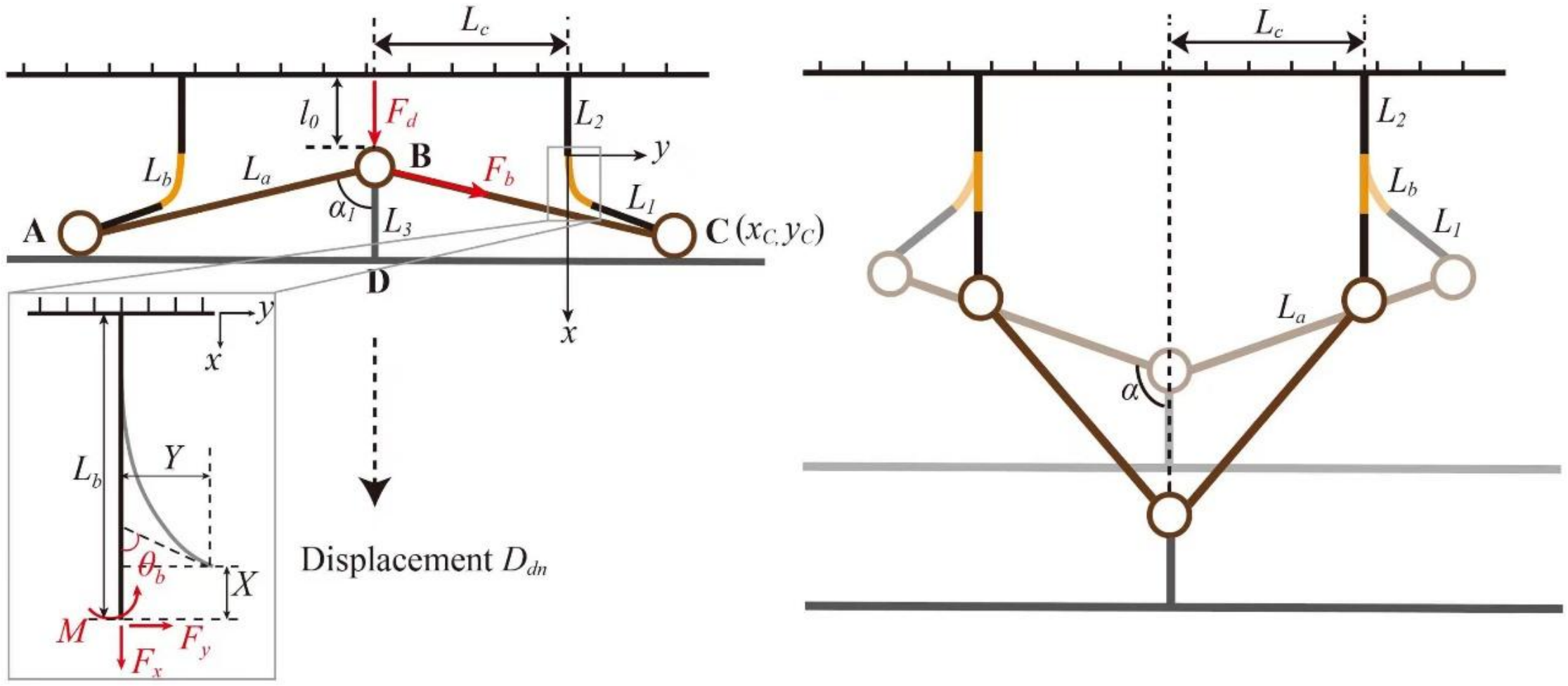

Fig. S2. Mechanical modeling and force analysis of the impulse module. The schematic illustrates the geometric relationships and kinematics of the mechanism during the impulsive deployment phase. Left: The initial state definition, where L_a and L_b represent the rigid link and flexible beam elements, respectively. The inset details the free-body diagram of the flexible beam L_b under large deflection, relating the coordinate forces (F_x, F_y) and bending moment (M) to the beam's deformation (X, Y). Right: The deformed state of the mechanism under the vertical displacement D_{dn} and driving force F_d, showing the evolution of the characteristic angle from α_1 to α.

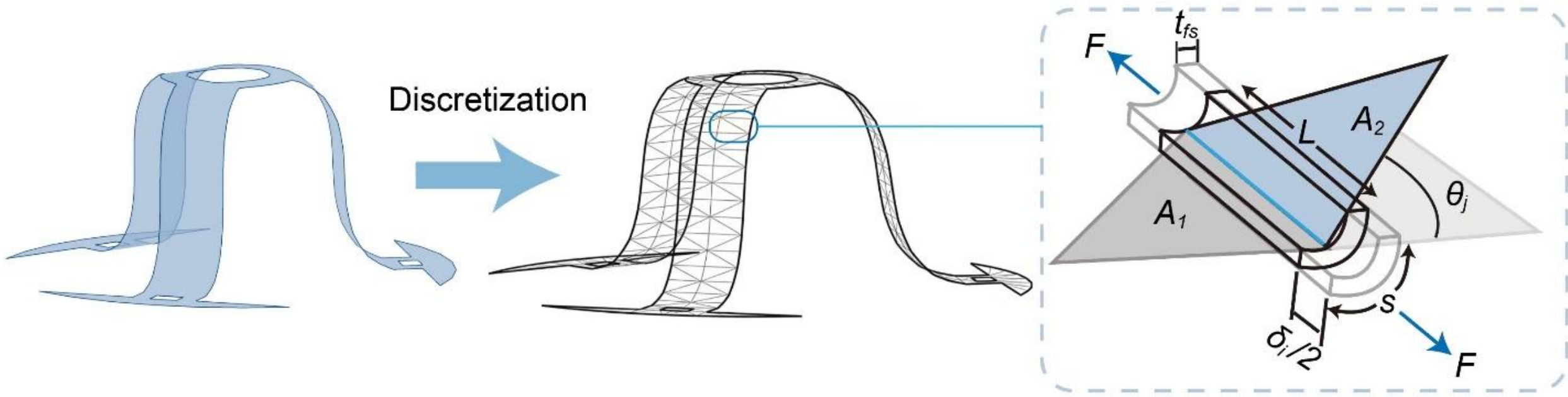

Fig. S3. Parameterized description of the bar-and-hinge unit cell. Left: Discretization of the continuous flexible sheet into a triangular mesh. Right: Geometric parameters (L, s, t_{fs}), effective areas (A_1, A_2) for the discretized rigid-bar element used in the theoretical model and δ_i is the corresponding extension of the bar. The two panels are rotated, relative to each other, by the hinge angle θ_j.

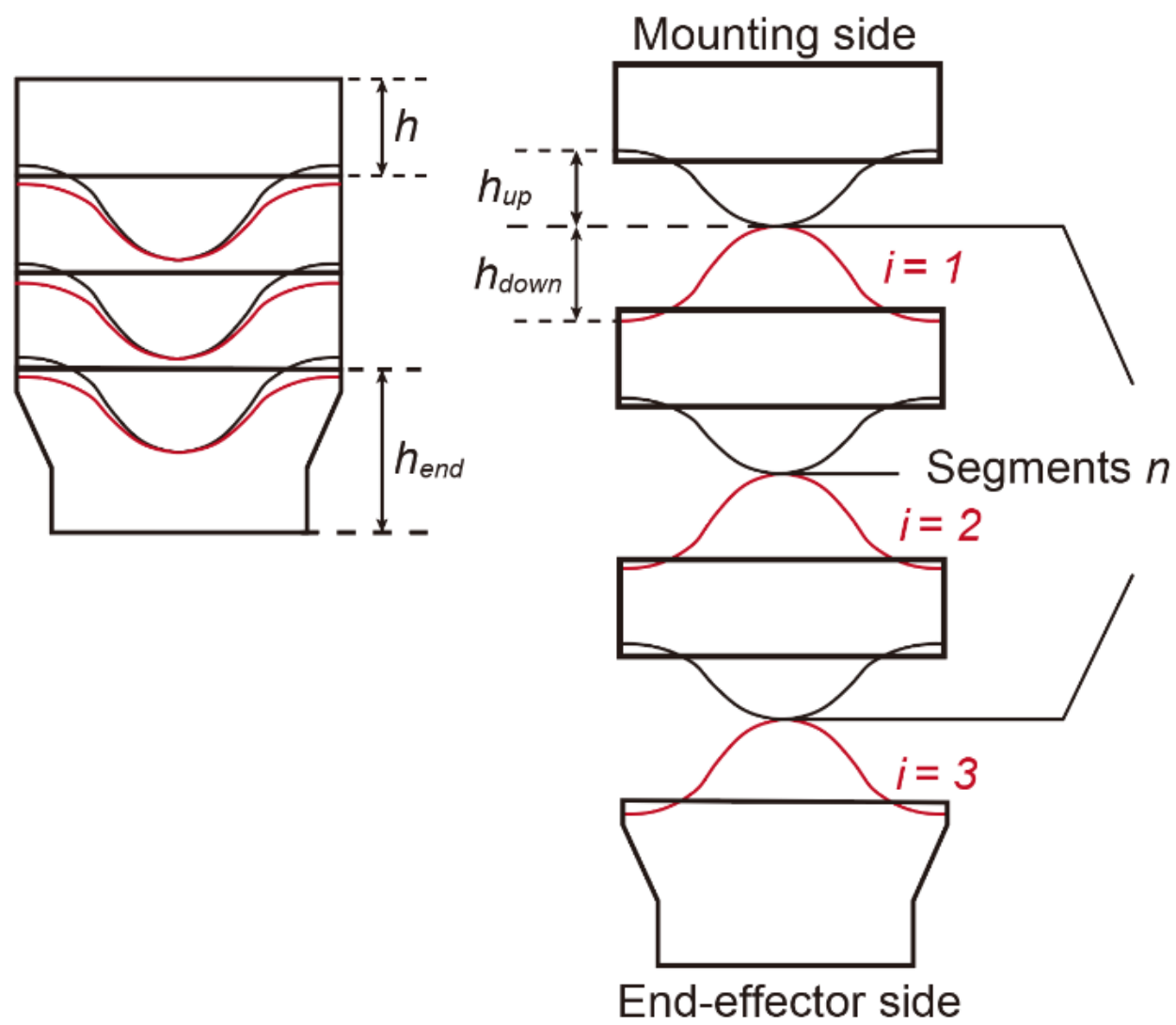

Fig. S4. Model parameters for extension ratio and energy analysis. The geometric variables h, h_{end}, h_{up}, and h_{down} are defined to characterize the deformation and extension behavior of the bistable arm. The left illustration shows the initial configuration of the arm, while the right illustration presents the downward-deployed configuration consisting of n serially connected bistable segments (here $n = 3$). Segment numbering starts from the UAV body and increases toward the end effector, with $i = 1$ denoting the segment closest to the UAV body.

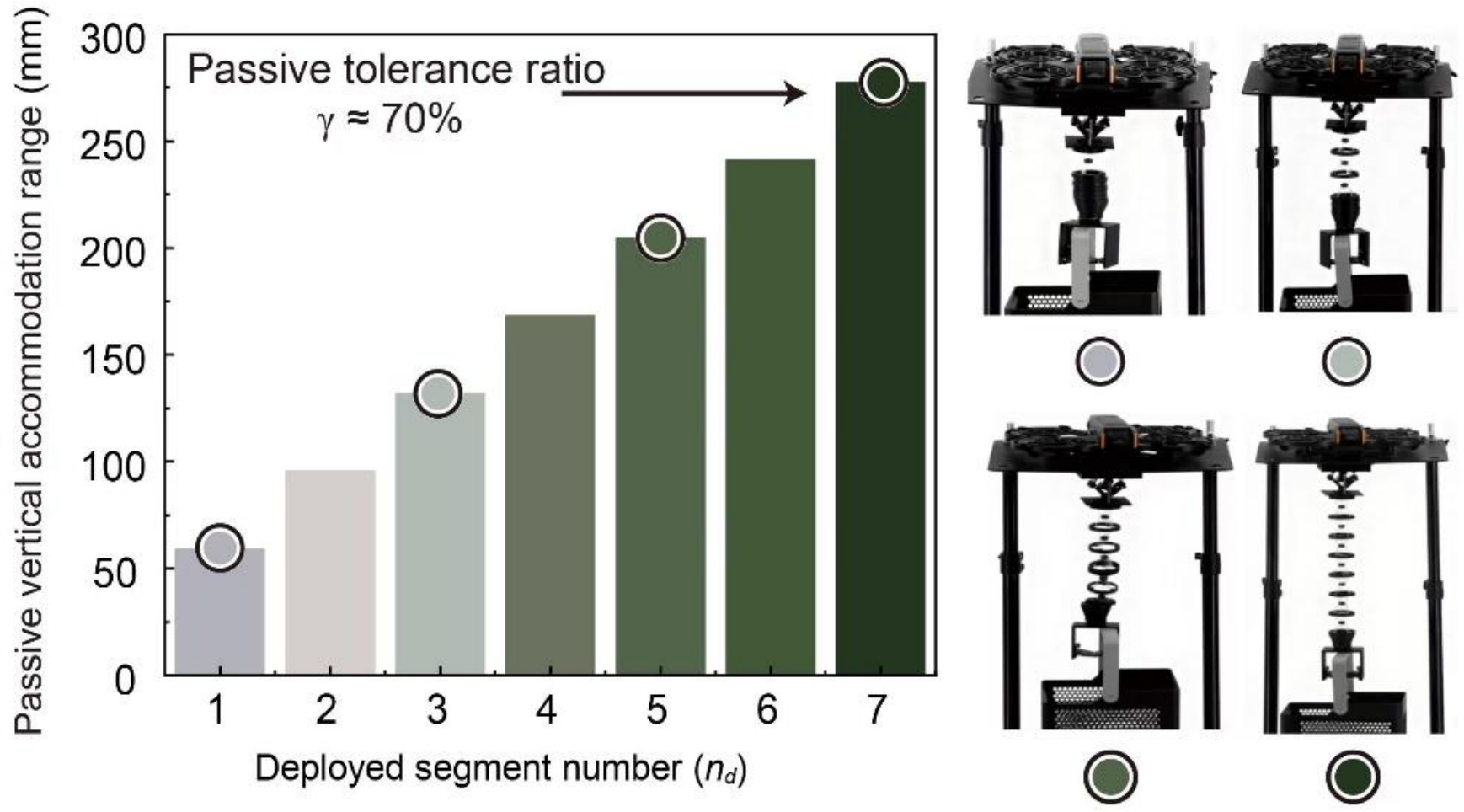

Fig. S5. Fixed-platform characterization of passive geometric tolerance in a seven-segment flexure arm. After activation, target contact constrained deployment at different deployed segment numbers n_d without active arm-length regulation. The measured vertical accommodation range increased with n_d. At $n_d = 7$, the passive vertical accommodation range ΔH was 277 mm, corresponding to approximately 70% of the fully extended arm length L_{el} of 395 mm.

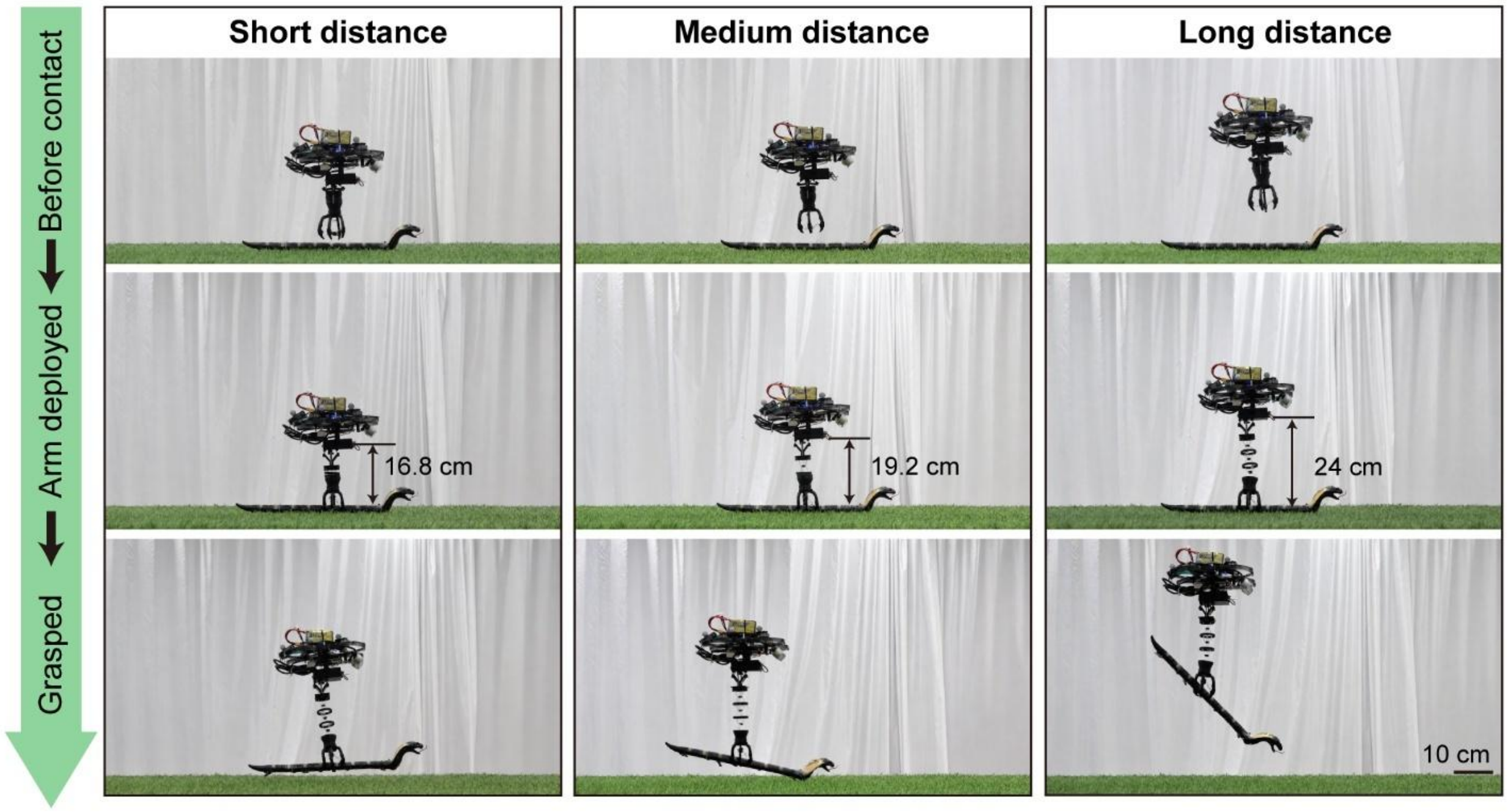

Fig. S6. Passive adaptation during aerial grasping at different distances between the UAV and target. Representative image sequences show grasping at short, medium, and long distances of 16.8, 19.2, and 24.0 cm, respectively. The top, middle, and bottom rows show the states before contact, after deployment and contact, and after grasping. The same three-segment arm configuration and triggering procedure were used in all three experiments. Active control positioned the UAV and initiated deployment. After triggering, target contact stopped further extension and passively determined the final deployed arm length, enabling grasping at all three distances.

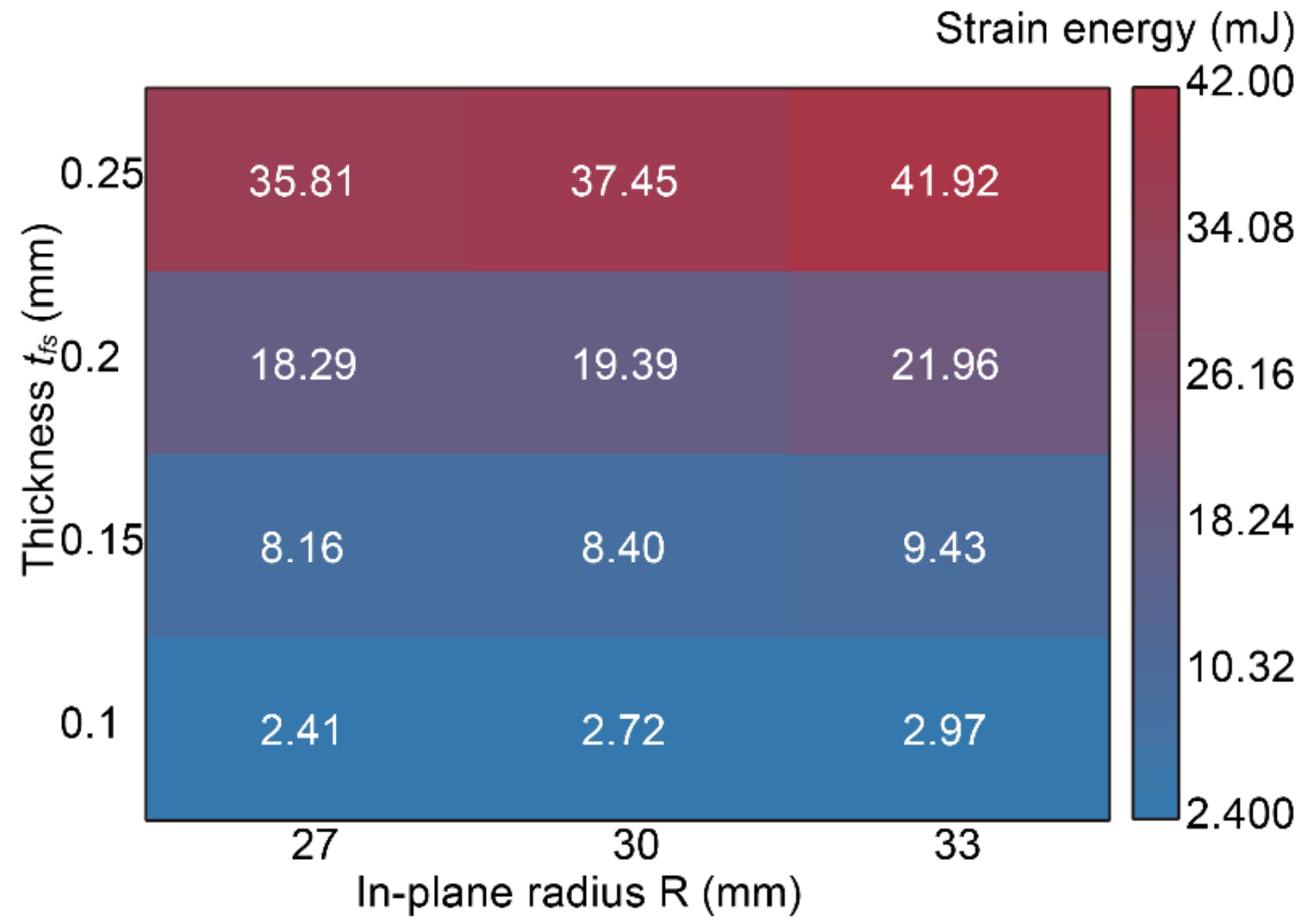

Fig. S7. Calculated strain energy of the bistable flexure unit. The heatmap shows strain energy for PET sheet thicknesses from 0.10 to 0.25 mm and in-plane radius from 27 to 33 mm. Each cell reports the calculated energy in mJ.

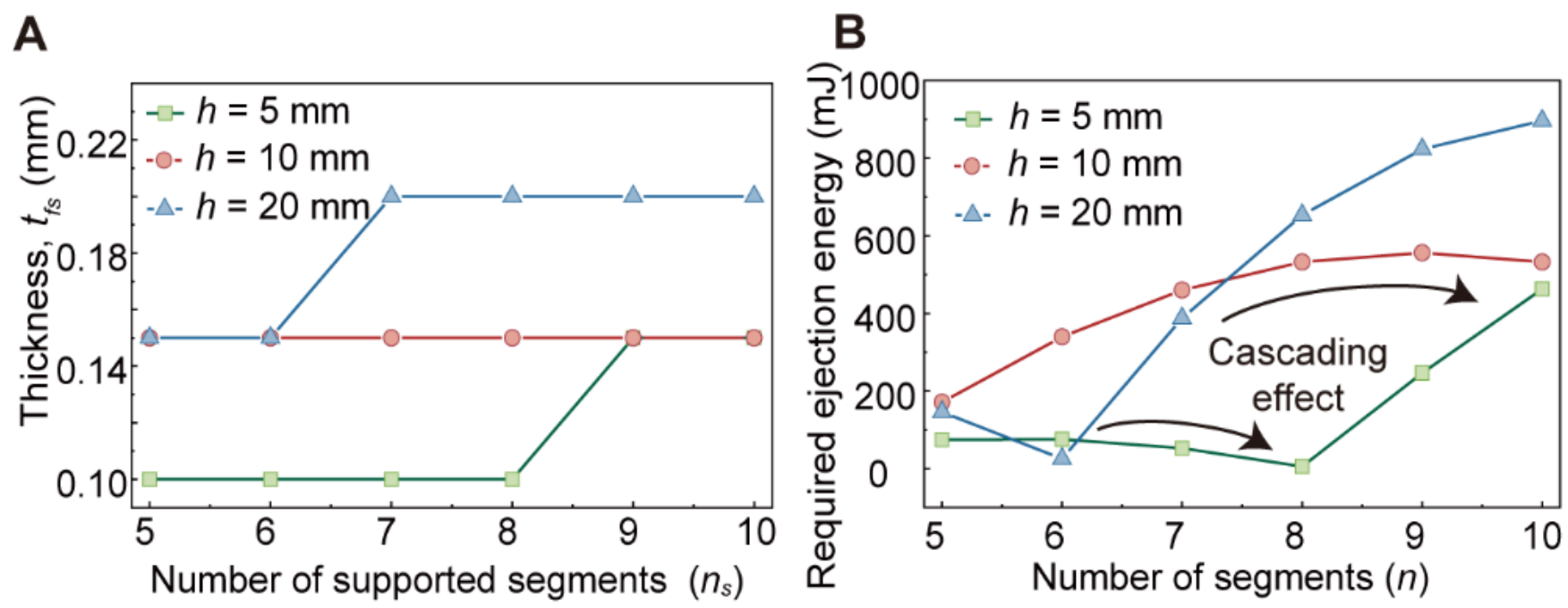

Fig. S8. Effects of holder height and arm configuration on the required sheet thickness and deployment energy. (A) Minimum PET flexible-sheet thickness t_{fs} required to prevent gravity-induced instability as a function of the supported-segment count n_s for holder heights of 5, 10, and 20 mm. Here, n_s denotes the number of segments supported by a given flexure unit, including the unit itself and all segments distal to it. For a downward-mounted arm, the lowest distal segment supports only itself ($n_s = 1$), while each successive segment toward the mounting side supports one additional segment. Under the proximal-to-distal position index i, $n_s = n + 1 - i$. (B) Required ejection energy as a function of the total segment number n during downward deployment. The nonmonotonic trends reflect the balance between gravity-assisted cascading and accumulated transmission losses.

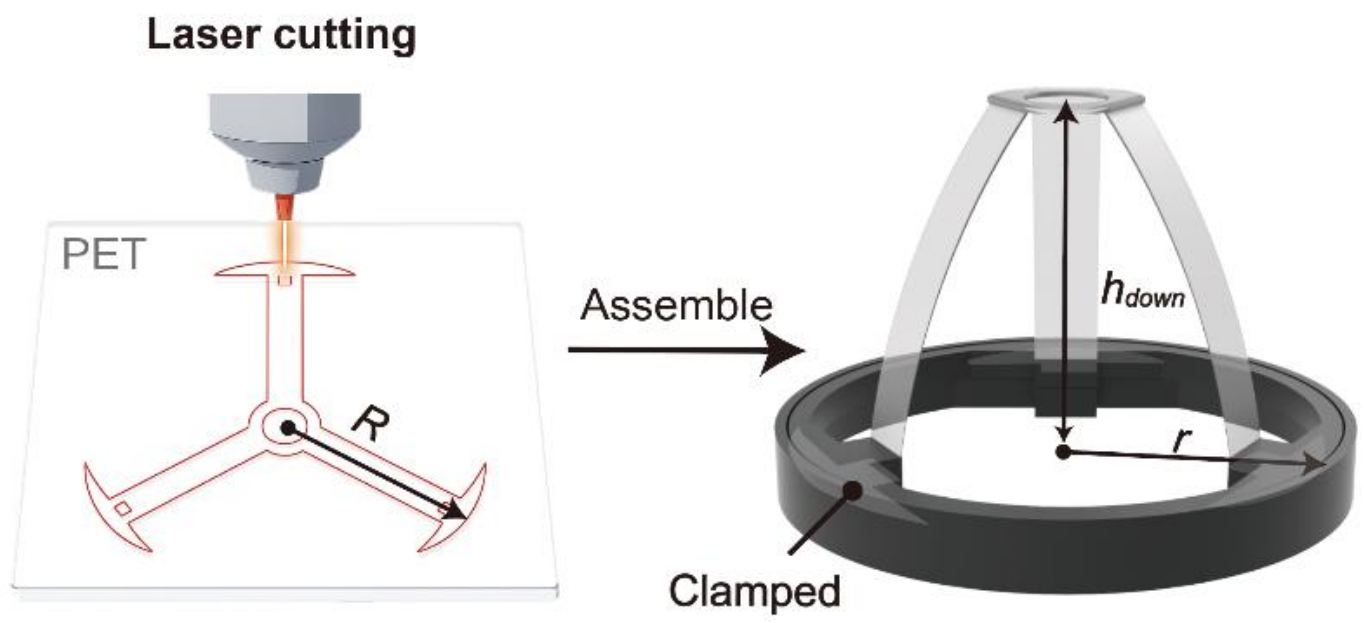

Fig. S9. Fabrication and assembly of a bistable flexure unit. The PET sheet is laser cut with an in-plane radius R and clamped into an annular holder with a deformed radius r. For the specified sheet topology, material, thickness, and clamping condition, the radial mismatch between R and r produces the out-of-plane bistable curvature and determines the assembled flexible-sheet height h_{down}.

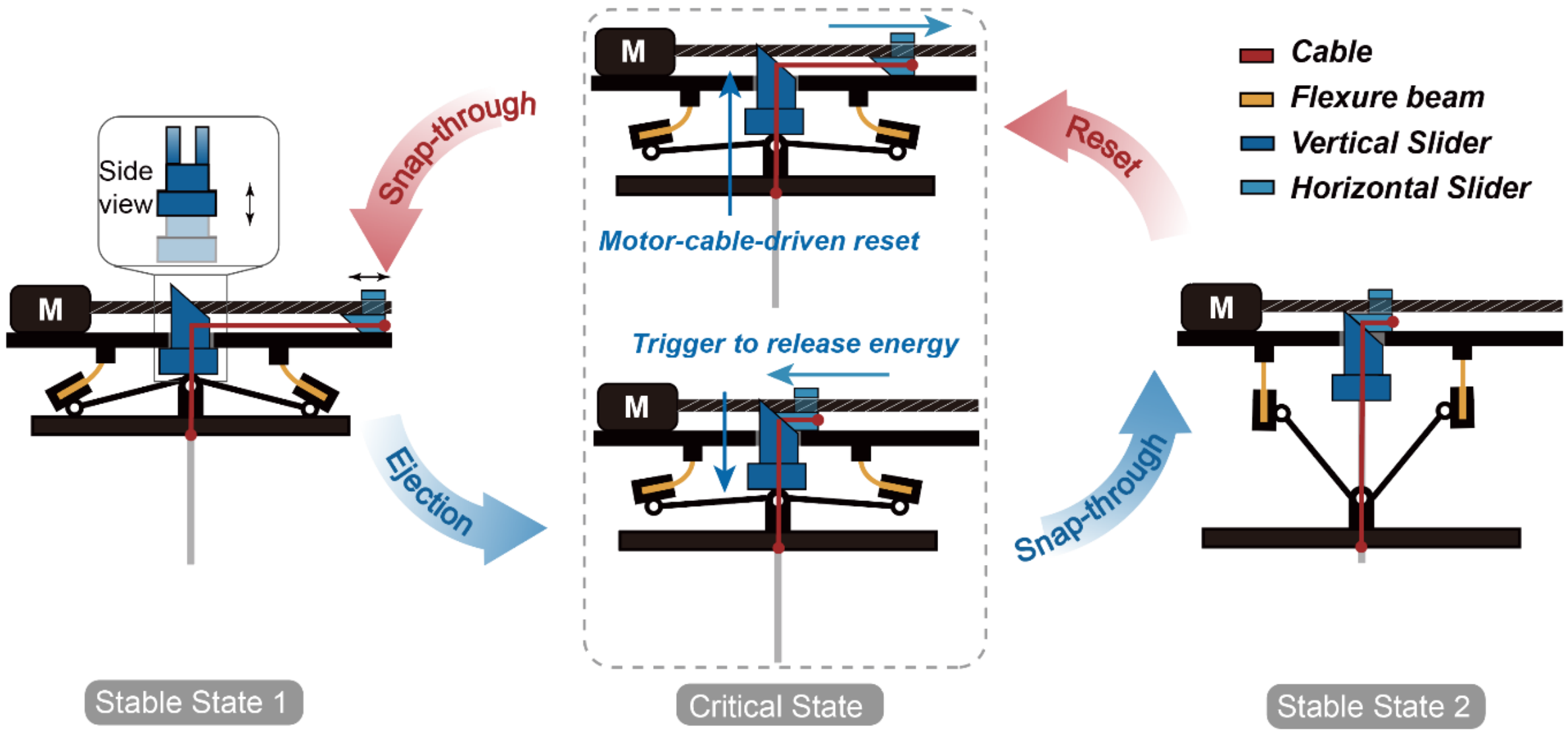

Fig. S10. Operating sequence of the asymmetric bistable impulse module. During ejection, the motor drives the horizontal slider from stable state 1 to the critical state and stores elastic energy in the mechanism. Trigger release initiates snap-through to stable state 2 and produces the impulse required for arm deployment. During reset, the motor-driven cable returns the mechanism to the critical state, followed by reverse snap-through to stable state 1.

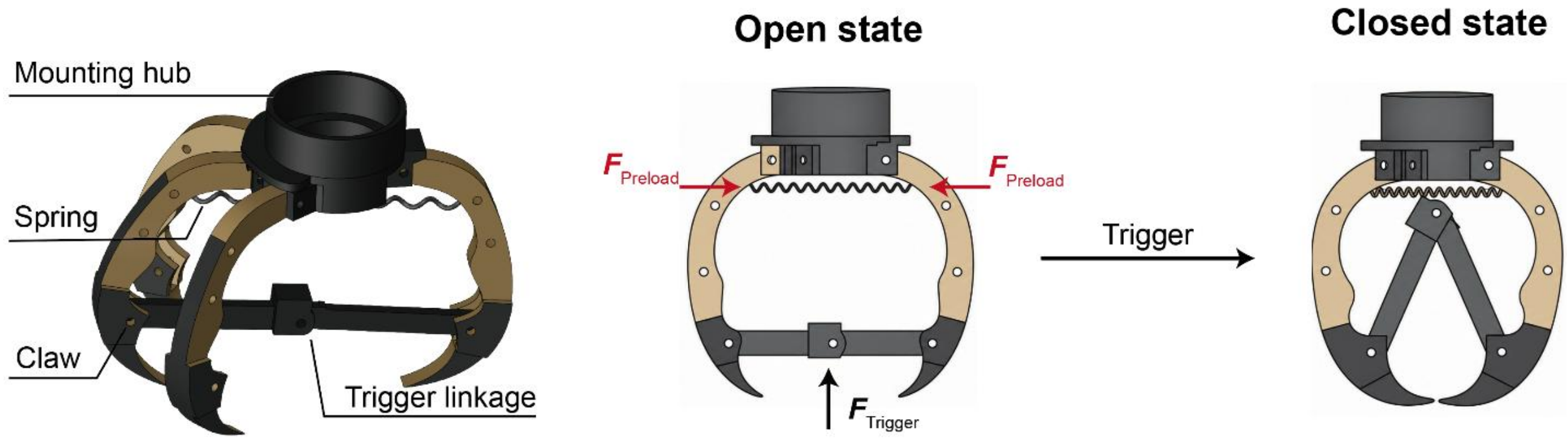

Fig. S11. Structure and contact-triggered operation of the passive gripper. The left panel identifies the mounting hub, preloaded spring, claws, and mechanical trigger linkage. The center and right schematics show the open and closed states, respectively. Target contact applies an upward force to the trigger linkage and releases the open-state constraint, allowing the stored spring energy to close the claws.

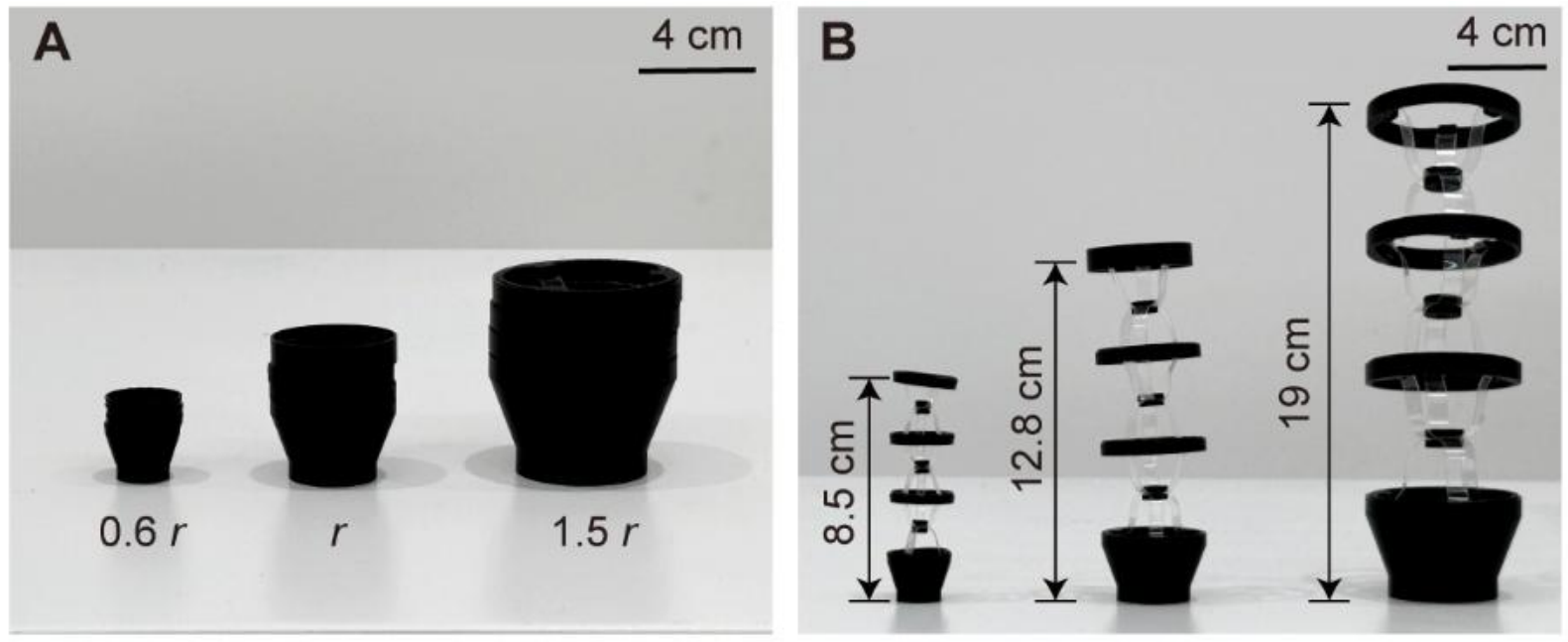

Fig. S12. Geometrically varied flexure arm prototypes. (A) End effectors with radii of 0.6r, r, and 1.5r. (B) Corresponding deployed configurations with arm lengths of 8.5, 12.8, and 19 cm.

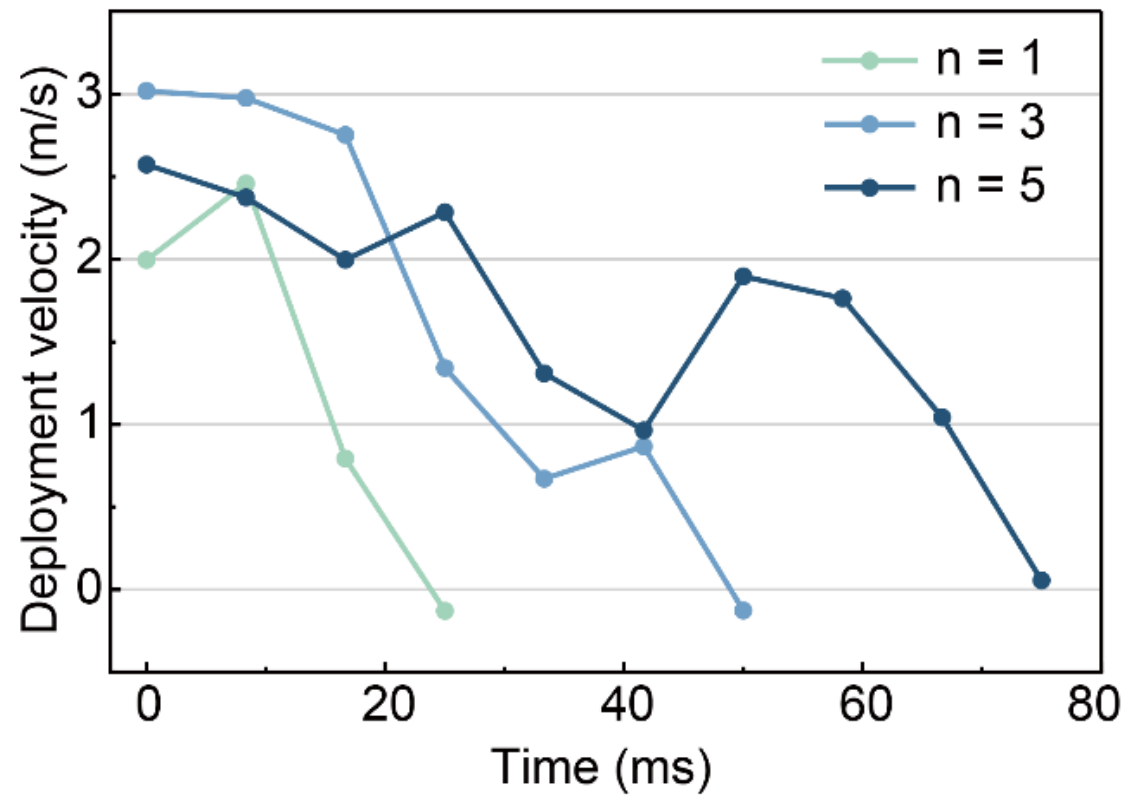

Fig. S13. Effect of segment number on deployment velocity. Deployment-velocity histories are shown for flexure arms containing $n = 1$, 3, and 5 segments. The corresponding deployment durations were approximately 25, 50, and 75 ms.

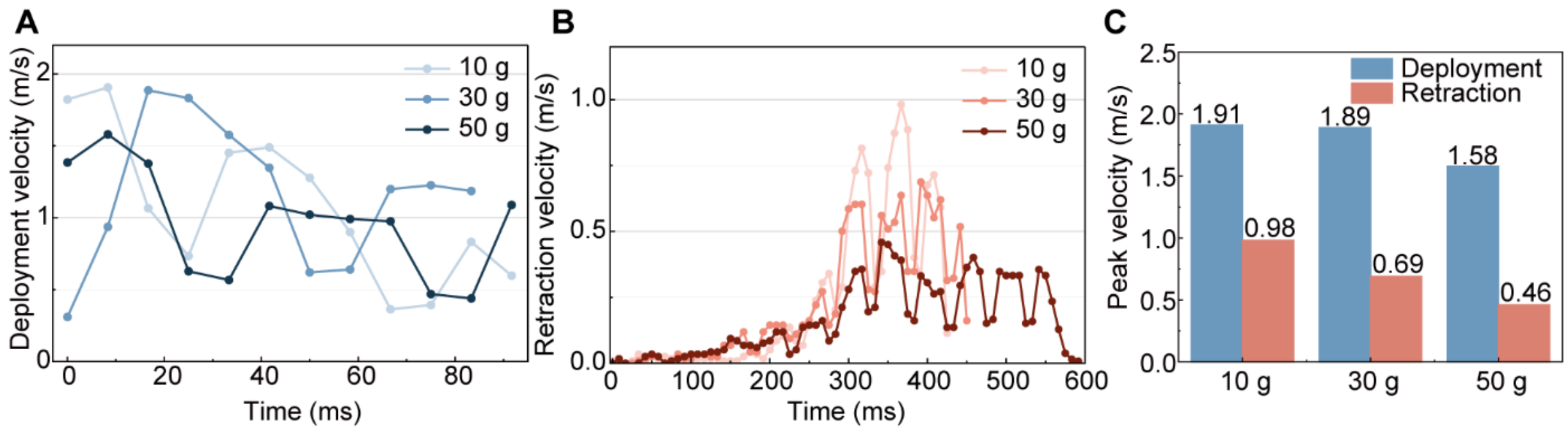

Fig. S14. Effect of payload mass on deployment and retraction velocity. (A) Deployment velocity and (B) retraction velocity for payloads of 10, 30, and 50 g. (C) Peak deployment and retraction velocities under the three payload conditions. The peak deployment velocities were 1.91, 1.89, and 1.58 m/s, and the corresponding peak retraction velocities were 0.98, 0.69, and 0.46 m/s. The retraction durations were 0.43, 0.45, and 0.62 s for payloads of 10, 30, and 50 g, respectively.

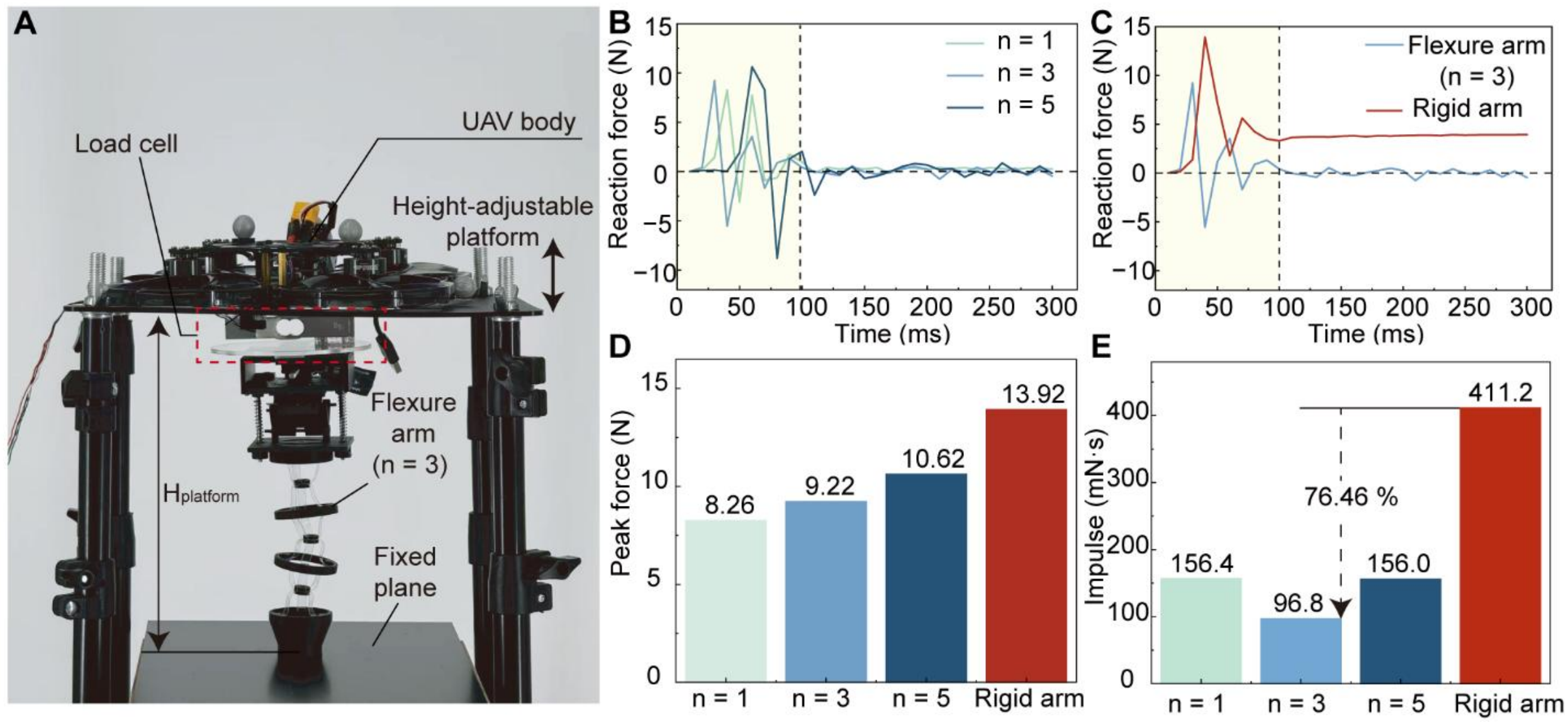

Fig. S15. Reaction loads measured at the UAV body during contact. (A) Test setup with the UAV body connected to a load cell through a height-adjustable platform while the arm contacts a fixed plane. (B) Reaction-force histories for flexure-arm configurations with n = 1, 3, and 5. (C) Comparison between the n = 3 flexure arm and the rigid arm at a matched contact height. The shaded regions in (B) and (C) indicate the first 100 ms following impulse-module triggering. (D) Peak reaction force and (E) impulse integrated over this interval for the tested configurations. At the matched contact height, the n = 3 flexure arm reduced the peak reaction force by 33.76% and the impulse by 76.46% relative to the rigid arm. For each test, $H_{platform}$ denoted in (A) was set to the fully deployed length of the corresponding arm configuration minus the same prescribed offset, ensuring contact with the fixed plane after deployment and a consistent comparison across configurations.

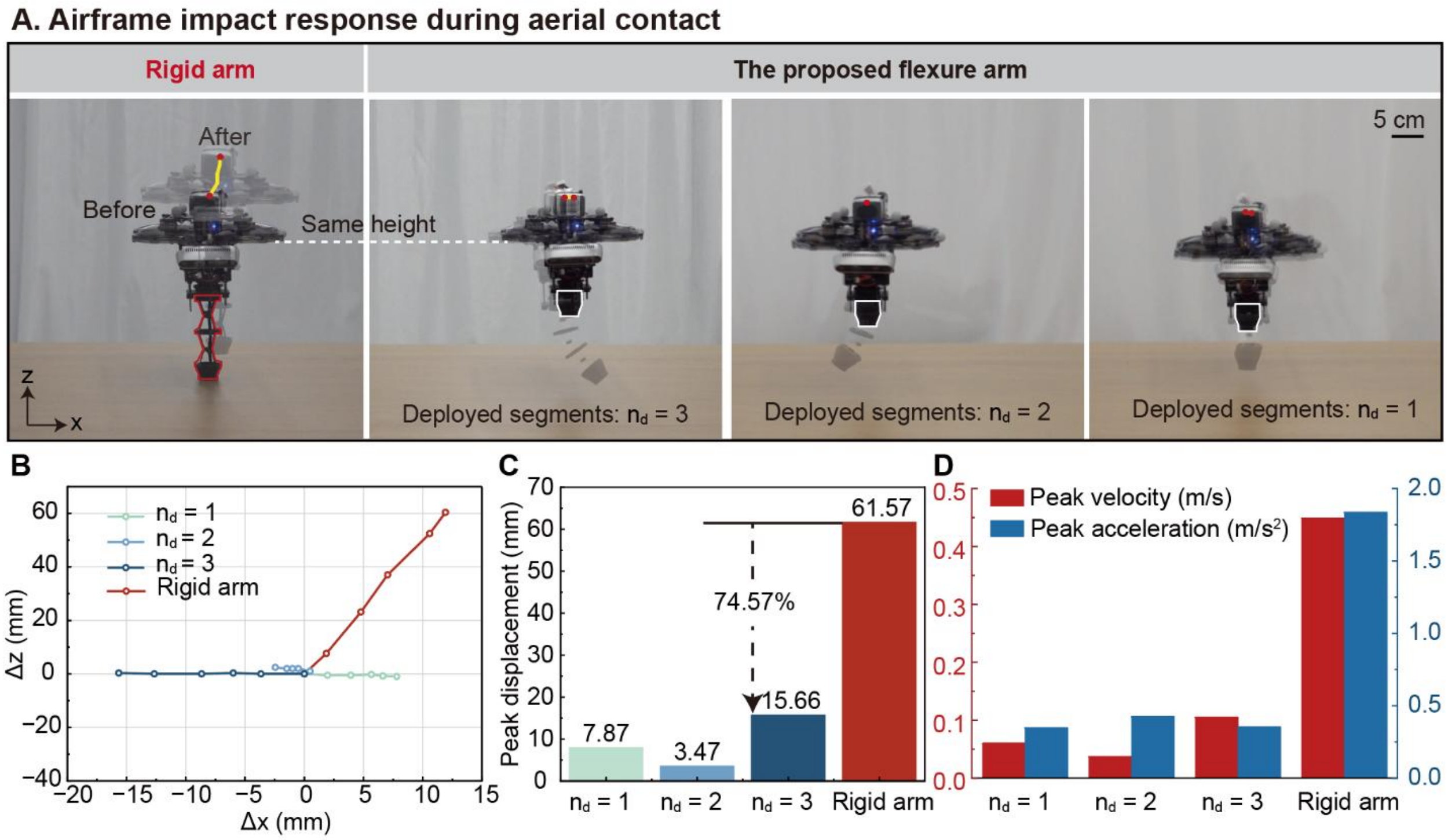

Fig. S16. Airframe impact response during aerial contact. (A) Images before and after contact for the rigid arm and for the flexure arm with n_d = 1, 2, and 3. The rigid arm and the n_d = 3 flexure arm were tested from the same initial UAV height. (B) Planar airframe displacement trajectories in the x-z plane. (C) Peak airframe displacement for each configuration. Under the matched-height condition, the n_d = 3 flexure arm reduced the peak displacement by 74.57% relative to the rigid arm. (D) Peak airframe velocity and acceleration for the same configurations. Both measures remained lower for the three tested flexure-arm configurations than for the rigid arm.

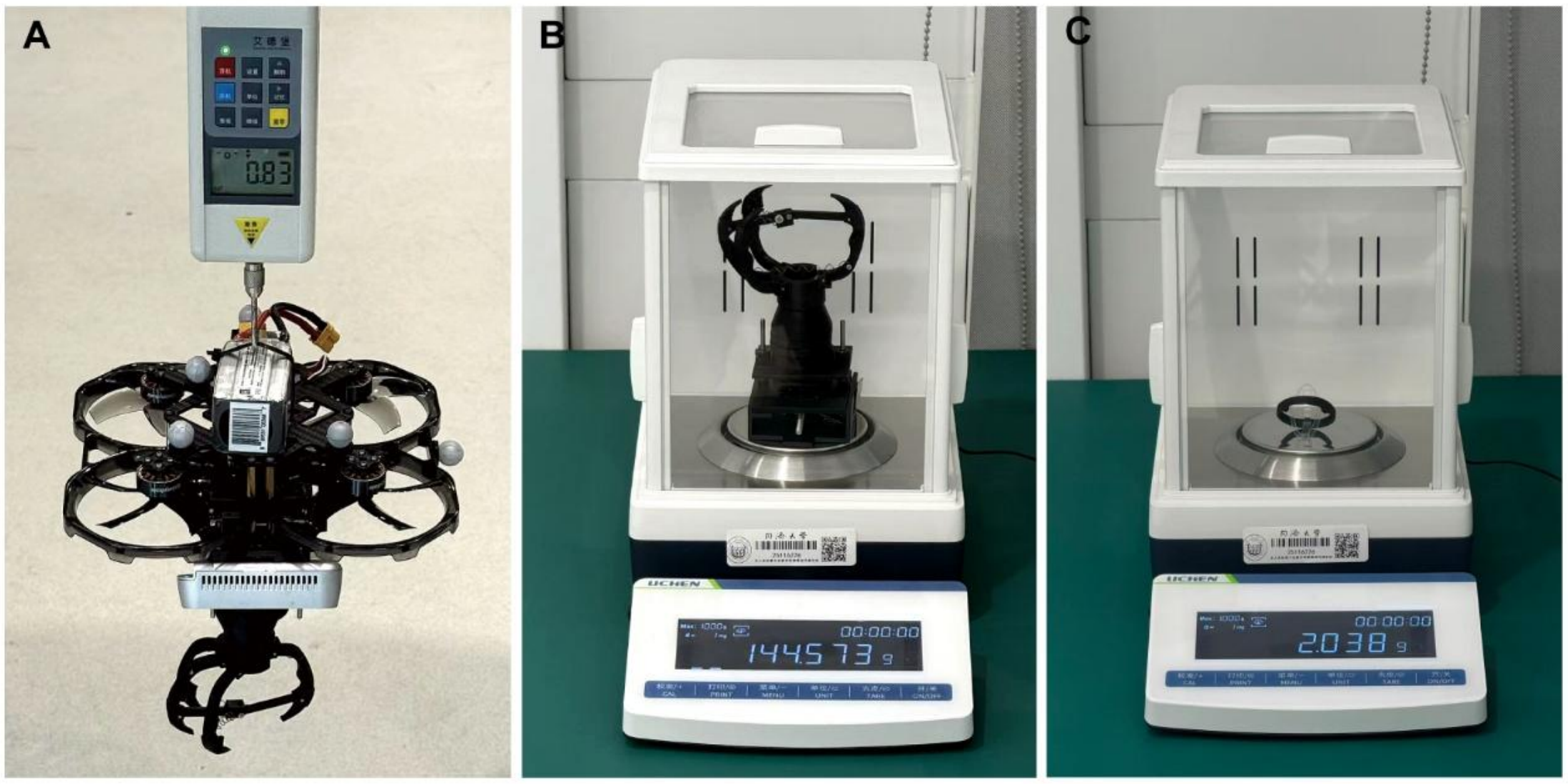

Fig. S17. Mass measurements of the aerial platform and limb components. (A) The integrated UAV and impulsive limb had a measured mass of 0.83 kg. (B) The complete impulsive limb assembly weighed 144.57 g. (C) A single assembled flexure unit weighed 2.04 g. The displayed readings correspond to the tested configuration.

Table S1. Geometric dimensions of the PET sheets forming the flexure arm. The arm is constructed from multiple bistable units, each consisting of a flexible sheet and a supporting sheet. The table details the specific dimensions used for downward-mounting and upward-mounting configurations.

Mounting Configuration	Component Role	In-plane Radius $\boldsymbol{R}$ (mm)	Deformed Radius $\boldsymbol{r}$ (mm)	Thickness $\boldsymbol{t_{fs}}$ (mm)
**Downward-mounting** *(Aerial Grasping)*	Flexible Sheet	30.0	19.2	0.15
	Supporting Sheet	35.0	19.2	0.25
**Upward-mounting** *(Perching)*	Flexible Sheet	30.0	19.2	0.05
	Supporting Sheet	35.0	19.2	0.25

Table S2. Nomenclature used in the main text and Supplementary Materials. Symbols are grouped by physical context. Superscripts identify coordinate frames, and subscripts identify bodies, targets, components, or indices where applicable.

Symbol	Definition	Symbol	Definition
**Impulse-module geometry and mechanics**			
L_1, L_2	Lengths of the lower and upper rigid links, respectively.	L_a, L_b	Lengths of the rigid side link and flexible beam, respectively.
L_3, L_c	Stopper length and auxiliary geometric length in the impulse-module model, respectively.	t_b	Thickness of the flexible beam in the impulse module.
X, Y	Flexible-beam tip displacements along the x and y axes, respectively.	x_C, y_C	Coordinates of point C in the simplified impulse-module geometry.
l_0, y_0	Initial hinge-to-plate distance and initial beam-tip vertical offset, respectively.	p	Parabolic-profile coefficient used to approximate the deflected beam shape.
θ_0, θ_b	Initial and current rotation angles of the flexible-beam free end, respectively.	α_1, α	Initial and instantaneous angles of the rigid side plate, respectively.
F_d, F_b	Applied downward force and force transmitted through the side plate, respectively.	F_x, F_y, M	Axial force, shear force, and bending moment at the flexible-beam free end.
D_{dn}, ξ	Downward tip displacement and integration coordinate used in the work calculation.	E_{ejection}	Elastic energy stored in the impulse module at the prescribed displacement.
E, G	Young's modulus and shear modulus of the flexible beam material.	A, I	Beam cross-sectional area and second moment of area, respectively.
**Flexure-arm geometry and bar-and-hinge model**			
R, r	In-plane radius of the laser-cut PET sheet and radius imposed by the annular holder.	t_{fs}	PET flexible-sheet thickness.
h, h_{end}	Intermediate holder height and end-holder height, respectively.	$h_{\text{down}}, h_{\text{up}}$	Heights of the lower and upper flexible sheets, respectively.
$L_{\text{rl}}, L_{\text{el}}$	Retracted and fully extended arm lengths measured between the same references, respectively.	ΔH	Passive vertical tolerance, defined as $\Delta H = L_{el} - L_{rl}$
k_e, γ	Ratio of the fully extended arm length to the retracted arm length, and passive vertical tolerance ratio, respectively.	n, n_d, n_s	n is the total number of flexure-arm segments, n_d is the number of deployed segments in the aerial-contact and geometric tolerance tests. n_s is the supported-segment count for a given flexure unit, including the unit itself and all segments distal to it. Under the proximal-to-distal indexing convention, $n_s = n + 1 - i$.
i, j	Indices for structural bars or arm units and virtual hinges, respectively.	δ_i, θ_j	Axial extension of the ith bar and rotation of the jth virtual hinge.
$U_{\text{total}}, U_{\text{bar}}, U_{\text{bending}}$	Total, in-plane bar, and hinge-bending strain energies.	k_s, k_b	Stiffness coefficients for bar extension and hinge bending.
L, A_T	Characteristic mesh length and area of one triangular panel.	A_1, A_2	Areas of the two triangular panels adjacent to a bending hinge.
D_{fs}	Tip displacement of the flexible sheet during mechanical characterization.	ρ_e	Arm extension rate, defined as $\rho_e = k_e/t_d$, with units of s^{-1}.
**Deployment-energy transmission and stability**			

Symbol	Definition	Symbol	Definition
E_{i+1}, η	Energy delivered to unit $i+1$ and inter-unit energy transmission efficiency, respectively.	$W_{g,i}$, $E_{\mathrm{left,i}}$	$W_{g,i}$ is the magnitude of the gravitational work associated with holder motion at unit i. Its contribution is added during downward deployment and subtracted during upward deployment.<br>$E_{\mathrm{left,i}}$ is residual mechanical energy after unit i completes snap-through.
ΔE_i	Energy difference between the two stable states of unit i.	G_i, m, g	Cumulative gravitational load, mass of one unit, and gravitational acceleration.
$F_{i,max}$	Maximum reverse snap-through force sustained by unit i.	t_i, R_i, r_i	Sheet thickness, in-plane radius, and holder radius of unit i.
**Perception, coordinate transformation, and approach control**			
$\{W\},\{B\},\{C\}$	World, UAV body, and camera coordinate frames, respectively.	$p_U^W, p_T^C, p_T^B, p_T^W$	UAV or target position expressed in the indicated coordinate frame.
R_{BC}, t_{BC}	Camera-to-body rotation matrix and camera-origin position in the body frame.	R_{WB}	Body-to-world rotation matrix obtained from the measured UAV pose.
X_U^W, Y_U^W, Z_U^W	Components of the UAV position in the world frame.	$x_{\mathrm{min}}, x_{\mathrm{max}}, y_{\mathrm{min}}, y_{\mathrm{max}}$	Pixel limits of the detected target bounding box.
u, v, d	Bounding-box center coordinates and aligned depth at the center pixel.	f_x, f_y, c_x, c_y	Camera focal lengths and principal-point coordinates in pixels.
$k, \Delta t$	Current sample index and sampling interval in the control model.	$v_T^W(\mathrm{k}), T_p$	Estimated target velocity and prediction horizon.
$p_{\mathrm{T,pred}}^W, p_{UT}^W$	Predicted target position and corresponding UAV trigger position.	$e_p^W(\mathrm{k})$	World-frame position error used by the outer-loop controller.
$v_{\mathrm{cmd,x}}^W, v_{\mathrm{cmd,y}}^W, v_{\mathrm{cmd,z}}^W$	Commanded UAV velocity components in the world frame.	$k_{p,i}, k_{d,i}$	Proportional and derivative gains along axis i.
d_{xy}, d_z	Horizontal alignment error and residual vertical offset, respectively.		
**Experimental configuration**			
H_{platform}	Platform height used to set the contact condition in the load-cell tests.	t_{imp}	Integration duration for the reaction-force measurements, set to 100 ms from impulse-module triggering.

Movie S1.

Autonomous in-flight interception of a moving target. This video demonstrates the complete autonomous mission pipeline, where the UAV detects, tracks, and intercepts a moving target (a snake replica). The onboard visual system estimates the target state in real-time, triggering the impulsive deployment once the target enters the system's geometric tolerance zone. The capture sequence is presented at 0.1× real-time speed to visualize the millisecond-level ballistic deployment of the morphological buffering framework.

Movie S2.

Versatile aerial physical interactions across unstructured environments. This video showcases the morphological intelligence across three distinct high-intensity scenarios: (1) Adaptive perching, where the limb secures an anchor on a branch, allowing the UAV to deactivate motors for energy-efficient long-duration monitoring; (2) High-impact barrier breaching (ice breaking), demonstrating the limb shattering an ice sheet while the flexure arm reduces the transmission of impact transients to the airframe (played at 0.1× speed); and (3) Cross-medium retrieval (air-to-water grasping), showing the ability to inject the end effector into water while the UAV remains above the water surface.

Movie S3.

Rapid viscous sampling (air to gel grasping) via a plunge-retract cycle. This video demonstrates the robotic limb's capability to operate in viscous media (gelatin). The system executes a rapid plunge-retract cycle to penetrate the medium at high speed (deployment: 0.04 s) and immediately retract against environmental resistance (retraction: 0.12 s).

Movie S4.

Reversible operation cycle: impulsive deployment and SMA-driven retraction. This video demonstrates the complete actuation cycle of the bio-inspired impulsive limb. Ballistic phase captures the high-speed impulsive deployment triggered by the snap-through instability of the impulse module, played at 0.1× real-time speed to visualize the ballistic extension kinematics. The recovery phase details the retraction process driven by the embedded SMA spring, played at 0.2× real-time speed to visualize the soft compression and resetting mechanics.

Movie S5.

Fixed-platform characterization of passive geometric tolerance in a seven-segment flexure arm. The seven-segment arm was allowed to deploy until target contact constrained it at different deployed segment numbers. The corresponding effective ranges shown for $n_d = 1, 3, 5,$ and 7 are 0 to 5.9 cm, 0 to 13.1 cm, 0 to 20.4 cm, and 0 to 27.7 cm, respectively. At $n_d = 7$, the effective range of 27.7 cm corresponds to approximately 70% of the fully extended arm length of 39.5 cm. In each trial, contact with the obstacle passively stopped further deployment without active arm-length regulation.

Movie S6.

Deployment with different segment numbers. This movie compares the impulsive deployment of flexure arms containing $n = 1$, 3, and 5 segments in real time and at 0.1× real-time speed, showing the sequential deployment of the modular bistable units.

Movie S7.

Reaction-force measurements during surface contact. This movie presents load-cell measurements of the transient reaction force transmitted to the UAV body. The first sequence compares the $n = 3$ flexure arm with the rigid arm at a matched contact height. The measured impulses over the first 100 ms following impulse-module triggering were 96.80 mN·s for the flexure arm and 411.20 mN·s for the rigid arm, corresponding to a reduction of 76.46%. The subsequent sequences show the reaction-force transients for flexure-arm configurations with $n =$ 1, 3, and 5.

Code S1.

Code for bar-and-hinge modeling of the bistable flexure unit. The package includes the main script, supporting functions, and an OBJ mesh file for calculating deformed configurations, force–displacement curves, and strain energy under prescribed boundary and loading conditions.
